# Experimental and Quantum Chemical Studies of Nicotinamide-Oxalic Acid Salt: Hydrogen Bonding, AIM and NBO Analysis

**DOI:** 10.3389/fchem.2022.855132

**Published:** 2022-03-15

**Authors:** Priya Verma, Anubha Srivastava, Poonam Tandon, Manishkumar R. Shimpi

**Affiliations:** ^1^ Department of Physics, University of Lucknow, Lucknow, India; ^2^ Department of Materials and Environmental Chemistry, Stockholm University, Stockholm, Sweden; ^3^ Chemistry of Interfaces, Luleå University of Technology, Luleå, Sweden

**Keywords:** nicotinamide–oxalic acid salt, spectroscopic signatures, hydrogen bonds, atoms in molecules, natural bond orbital, reactivity–property study

## Abstract

The computational modeling supported with experimental results can explain the overall structural packing by predicting the hydrogen bond interactions present in any cocrystals (active pharmaceutical ingredients + coformer) as well as salts. In this context, the hydrogen bonding synthons, physiochemical properties (chemical reactivity and stability), and drug-likeliness behavior of proposed nicotinamide–oxalic acid (NIC–OXA) salt have been reported by using vibrational spectroscopic signatures (IR and Raman spectra) and quantum chemical calculations. The NIC–OXA salt was prepared by reactive crystallization method. X-ray powder diffraction (XRPD) and differential scanning calorimetry (DSC) techniques were used for the characterization and validation of NIC–OXA salt. The spectroscopic signatures revealed that (N7–H8)/(N23–H24) of the pyridine ring of NIC, (C═O), and (C–O) groups of OXA were forming the intermolecular hydrogen bonding (N–H⋯O–C), (C–H⋯O═C), and (N–H⋯O═C), respectively, in NIC–OXA salt. Additionally, the quantum theory of atoms in molecules (QTAIM) showed that (C10–H22⋯O1) and (C26–H38⋯O4) are two unconventional hydrogen bonds present in NIC–OXA salt. Also, the natural bond orbital analysis was performed to find the charge transfer interactions and revealed the strongest hydrogen bonds (N7–H8⋯O5)/(N23–H24⋯O2) in NIC–OXA salt. The frontier molecular orbital (FMO) analysis suggested more reactivity and less stability of NIC–OXA salt in comparison to NIC–CA cocrystal and NIC. The global and local reactivity descriptors calculated and predicted that NIC–OXA salt is softer than NIC–CA cocrystal and NIC. From MESP of NIC–OXA salt, it is clear that electrophilic (N7–H8)/(N23–H24), (C6═O4)/(C3═O1) and nucleophilic (C10–H22)/(C26–H38), (C6–O5)/(C3–O2) reactive groups in NIC and OXA, respectively, neutralize after the formation of NIC–OXA salt, confirming the presence of hydrogen bonding interactions (N7–H8⋯O5–C6) and (N23–H24⋯O2–C3). Lipinski’s rule was applied to check the activeness of salt as an orally active form. The results shed light on several features of NIC–OXA salt that can further lead to the improvement in the physicochemical properties of NIC.

## 1 Introduction

Pharmaceutical salts and cocrystals represent a particularly interesting and relevant group of multicomponent solids. Cocrystal is composed of neutral species, and salt contains cation and anion (Aitipamula et al., 2012). Sometimes, it is challenging to establish if a proton has been fully transferred from an acid to a base. Consequently, if the goal is to synthesize multicomponent solids, i.e., cocrystal, supramolecular synthon prediction is an advantage and probably more important from a patent perspective.

In the pharmaceutical industry, more than 40% of active pharmaceutical ingredients (APIs) in phase III (clinical trials) fail due to problems associated with solubility or less effective bioavailability as compared to currently used drugs. The major challenge among scientists working in pharmaceutical solid materials is to modify the physical and chemical properties like stability, crystallinity, solubility, and bioavailability of APIs (Yadav et al., 2009). The salt formation leads to the enhancement in physiochemical properties of ionizable pharmaceutical ingredients (APIs) that are applicable for ionizable drug molecules (Acharya et al., 2018). In the cocrystal approach, supramolecular assemblies that are comprised of homo- and heterosynthons play pivotal roles in designing and developing drugs that rely on hydrogen bonds and are applicable for ionizable as well as non-ionizable drug molecules. The factors influencing packing geometry proposed that classical (weak) as well as non-classical (strong) intermolecular hydrogen bonds are useful to understand the properties and behavior in crystals (Desiraju, 1995). Theoretically, density functional theory (DFT) calculations coupled with spectroscopic analysis are used to determine and validate the structure–property relationship of molecules (Liu et al., 2018).

NIC (pyridine-3-carboxamide), vitamin B_3_, is used to cure pellagra, dementia, and inflammatory diseases. Most recent studies have proved the therapeutic nature of this drug (Gasperi et al., 2019). Oxalic acid (OXA) belongs to the family of carboxylic acid and is hydrophilic in nature, which can be produced by animals, plants, bacteria, and fungi (Hodgkinson and Zarembski, 1968). In the carboxylic acid (COOH) functional group, both donor (OH) and acceptor (C═O) moieties are present and have a tendency of forming strong hydrogen bonds with heteroaromatic rings of a nitrogen atom (Childs and Hardcastle, 2007; Verma et al., 2021). The structural analysis of nicotinamide–OXA (NIC–OXA) salt revealed the layered structure in which two NIC cations and one oxalate ion interact *via* strong and weak hydrogen bonds N^+^–H⋯O^−^ and C–H⋯O, respectively (Shimpi et al., 2017).

To the best of our knowledge, no studies have been explored to examine the stability and structure–reactivity–property relationship of NIC and NIC–OXA salt by using spectroscopy and theoretical approaches. In continuation of our previous work on nitrofurantoin-4-dimethylaminopyridine (NF-DMAP) salt (Khan et al., 2019) and NIC–citric acid (NIC–CA) cocrystal (Verma et al., 2019), the current study is focused on the prediction of physicochemical properties of NIC–OXA salt and its comparison with NIC–CA cocrystal (Verma et al., 2019). Due to this fact, the ground-state optimized structure, hydrogen bond motifs, their energies, and structure–reactivity relationship of NIC–OXA salt have been reported by DFT calculations (Varbanov et al., 2013). The IR and Raman spectra of NIC–OXA salt have been plotted using B3LYP/6-311++G(d,p) methodology and compared with experimental evidence (Shimpi et al., 2017). X-ray powder diffraction (XRPD) and differential scanning calorimetry (DSC) method has been used to analyze and identify the presence of crystalline phases of NIC–OXA product material. The strength and nature of bonds involved in hydrogen bonding have been elaborated by Bader’s quantum theory of atoms in molecules (QTAIM) (Kumar et al., 2016). Further, natural bond orbital (NBO) analysis (Dunnington and Schmidt, 2012) provided the resultant stabilization parameters responsible for the formation of NIC–OXA salt. The frontier molecular orbital (FMO) analysis and chemical reactivity parameters, viz., global reactivity, predicted the various aspects of structure–stability, electrophilicity, and electronic state properties. Additionally, local reactivity descriptors such as Fukui function, local softness, and electrophilicity index have been suggested to have interactive trends of molecules. The drug-like properties of NIC–OXA salt have been studied by Lipinski’s rule of five (Lipinski et al., 1997).

## 2 Experimental Details

Reaction crystallization (Rodríguez-Hornedo et al., 2006) or reactive crystallization (McDonald et al., 2021) was used to prepare the NIC–OXA salt. Reaction crystallization causes high local supersaturation resulting in fast nucleation rates for product formation. Such a technique is promoted as using a larger-scale crystallization process as well as being environmentally friendly. For the reaction crystallization, a 4-ml glass vial was charged with 244.1 mg (2 mmol) of NIC and 180 mg (2 mmol) of OXA. About 2 ml of trihexyl(tetradecyl)phosphonium bis(oxalato)borate, (P_6,6,6,14_) (BOB), and ionic liquids were added to form a slurry. The mixture was constantly stirred for a total of 8 days, at which time the solids present were isolated by vacuum filtration and the ionic liquid was removed using toluene. Further, the solid white product was air-dried at room temperature. Prepared salt was analyzed using XRPD and DSC to validate the product material, and data are shown in Supplementary Figures S1, S2, respectively.

The IR spectrum was recorded on a Bruker Vertex 80v Fourier transform IR (FT-IR) spectrometer equipped with a DLaTGS detector and a Platinum-ATR accessory with a diamond crystal as an ATR element. Both a single beam background and spectra without and with the powered samples, respectively, were obtained by averaging 128 scans with an optical resolution of 4 cm^−1^. A spectrum was recorded under vacuum using the double-side forward–backward acquisition mode. Raman spectrum was recorded using a Raman microscope (Kaiser Optical Systems, Inc., Ann Arbor, MI, United States) with 785-nm laser excitation. The laser power at the solid sample was approximately 100 mW. Spectrum was obtained for one 10-s exposure of the charge-coupled device (CCD) detector in the wavenumber range 100–4,000 cm^−1^. The XRPD pattern for the sample was collected using an Empyrean X-ray diffractometer (PANalytical, Almelo, Netherlands) equipped with a PIXel3D detector and a monochromatic CuKα*1* radiation X-ray tube (*λ* = 1.54056 Å). The tube voltage and ampere were set at 45 kV and 40 mA, respectively. The white product solid sample was scanned 2θ range of 5°–40°, increasing at a step size of 0.02. Thermal analyses of samples were performed on a TA Instruments DSC Q1000. Samples (1–2 mg) were crimped in non-hermetic aluminum pans and scanned at a heating rate of 10°C/min under a continuously purged dry nitrogen atmosphere (flow rate 50 ml/min) using a similar empty pan as a reference.

## 3 Quantum Chemical Calculations and Computational Details

All calculations were performed by DFT (Perdew and Ruzsinszky, 2010) approach along with B3LYP/6-311++G(d,p) methodology (Parr, 1980; Lee et al., 1988; Becke, 1993) using the Gaussian09 program (Frisch, 2009). The optimized structures, their energies, and geometries obtained by the Gaussian output file were visualized by GaussView (Frisch et al., 2000) program. The Raman scattering amplitudes cannot be directly taken as intensity obtained by DFT calculations (Guirgis et al., 2003). So Raman scattering cross-section (∂σ_j_/∂Ω), which is directly proportional to intensity, was calculated from predicted vibrational wavenumbers for each of the normal modes (Polavarapu, 1990). The spectroscopic (IR and Raman) signatures of NIC, OXA, and NIC–OXA salt were obtained by the Gar2Ped program (Martin and Van Alsenoyand Gar2ped, 1995) based on potential energy distribution (PED) and Pulay’s recommendations (Pulay et al., 1979). The obtained wavenumber values were scaled by linear scaling procedure wavenumber-linear scaling (WLS) (Yoshida et al., 2002) to add anharmonicity effects in the vibrations by

νobs=(1.0087−0.0000163υcal)υcal cm−1

Topological parameters of bonds at bond critical points (BCPs) of interacting atoms were studied by using AIM 2000 (Bader and Cheeseman, 2000) and AIM ALL program packages (Keith and AIMALL, 2009). The NBO calculations were performed at the same theory level as geometry optimization.

The global reactivity descriptors like hardness (η), electronegativity (χ), chemical potential (μ), softness (S), and electrophilicity index (ω) were given by highest occupied molecular orbital (HOMO)–lowest unoccupied molecular orbital (LUMO) energies of molecules by the formulae as (Parr and Pearson, 1983; Geerlings et al., 2003; Chattaraj and Roy, 2007)

$$\eta =\;\frac{1}{2}\left(E_{\mathrm{LUMO}}-E_{\mathrm{HOMO}}\right)$$

$$\chi =\;-\frac{1}{2}\left(E_{\mathrm{HOMO}}+\;E_{\mathrm{LUMO}}\right)$$

$$\mu =\;-\chi =\;\frac{1}{2}\left(E_{\mathrm{HOMO}}+E_{\mathrm{LUMO}}\right)$$

$$S=\;\frac{1}{2\eta }$$

$$\omega =\;\frac{\mu^{2}}{2\eta }$$

The maximum electronic charge ( ${\text{ΔN}}_{\text{max}}$ ) gained by an electrophile from the surroundings is expressed as

$$\Delta N_{\max}=\;-\frac{\mu }{\eta }$$

The electrophilic charge transfer (ECT) (Padmanabhan et al., 2007) is the difference between ∆N_max_ values of two interacting molecules X and Y expressed as

$$\mathrm{ECT}={\left(\Delta N_{\max}\right)}_{X}-{\left(\Delta N_{\max}\right)}_{Y}$$

where ${\left(\Delta N_{\max}\right)}_{X}=\;-\frac{\mu_{X}}{\eta_{X}}$ and ${\left(\Delta N_{\max}\right)}_{Y}=\;-\frac{\mu_{Y}}{\eta_{Y}}$ .

If ECT value >0, the charge will flow from molecule Y to X. Similarly, if ECT < 0, the charge will flow from molecule X to Y (Padmanabhan et al., 2007).

The condensed Fukui functions like ( ${\text{f}}_{\text{k}}^{+},{\text{s}}_{\text{k}}^{+},{\text{ω}}_{\text{k}}^{+}$ ), ( ${\text{f}}_{\text{k}}^{-},{\text{s}}_{\text{k}}^{-},{\text{ω}}_{\text{k}}^{-}$ ), and ( ${\text{f}}_{\text{k}}^{0},{\text{s}}_{\text{k}}^{0},{\text{ω}}_{\text{k}}^{0}$ ) can be used to define the nucleophilic, electrophilic, and radical attacks, respectively (Li and Evans, 1995).

Molar refractivity (MR) value is a quantitative structure–activity relationship (QSAR) (Sawale et al., 2016) for a drug molecule, given by Lorentz–Lorenz formula (Padrón et al., 2002; Verma and Hansch, 2008) as

$$\text{MR}=\left[\frac{{\text{n}}^{2}-1}{{\text{n}}^{2}+2}\right]\left(\frac{\text{MW}}{\text{ρ}}\right)=1.333\text{πNα}$$

where n, MW, ρ, (MW/ρ), α, and N are the refractive index, molecular weight, density, molar volume, polarizability, and Avogadro’s number, respectively, of a molecule.

## 4 Results and Discussion

### 4.1 Geometry Minimization and Energies

**FIGURE 1:**
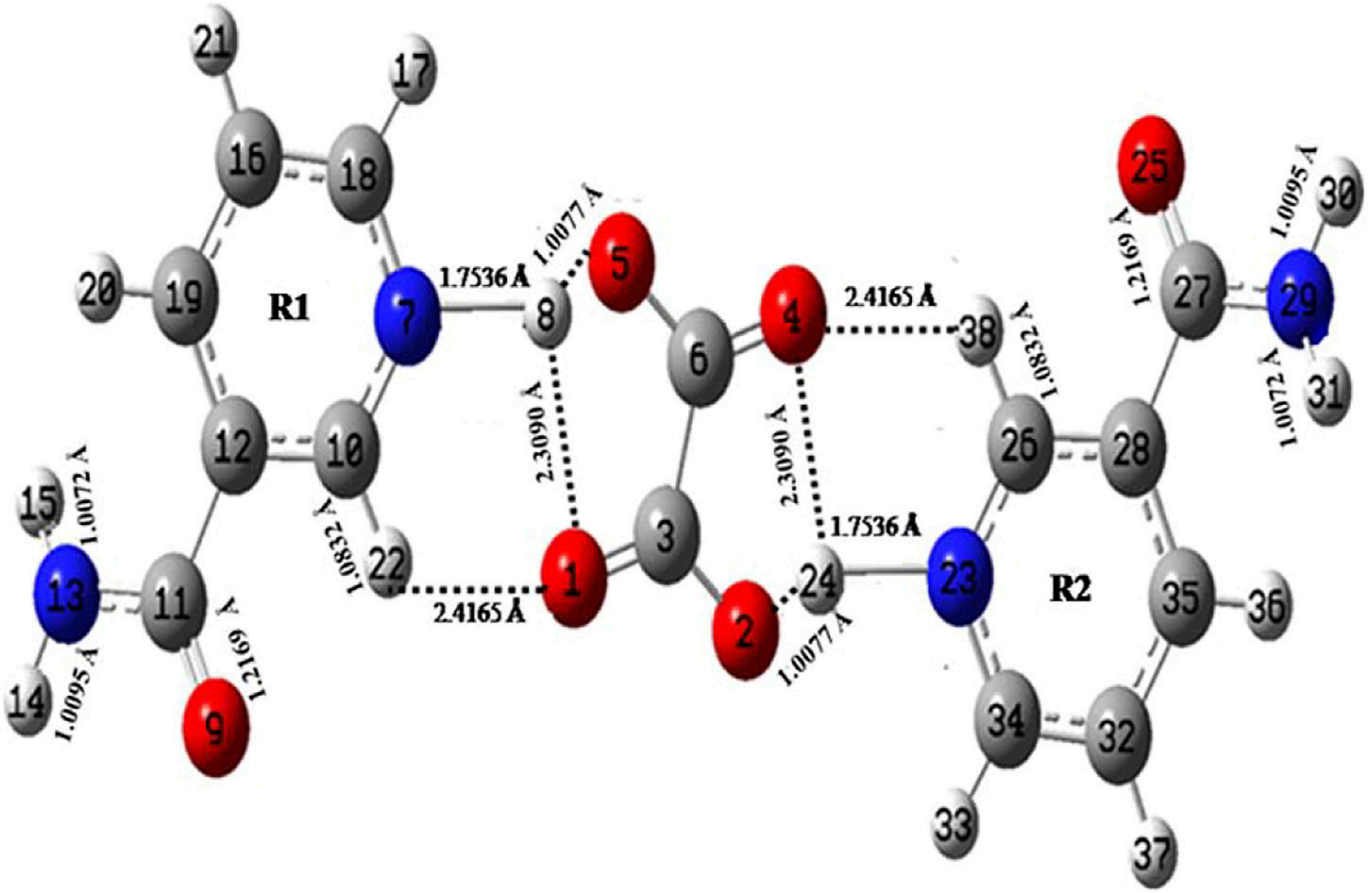
Optimized structure of NIC–OXA salt with atom numbering adopted in this study. NIC–OXA, nicotinamide–oxalic acid.

The crystal structures of NIC, OXA, and NIC–OXA salt have been reported, and the initial geometries were obtained from their respective crystallographic data (Jarzembska et al., 2014; Shimpi et al., 2017; Bhattacharya, 2020). The obtained ground state (minimized) structures with the atomic numbering of NIC, OXA, and NIC–OXA salt are given in Supplementary Figures S3, S4, and Figure 1, respectively. The crystal structures of NIC, OXA, and NIC–OXA salt with hydrogen bonding network are shown in Supplementary Figures S5–S7, respectively.

The optimized geometrical parameters (bond lengths, bond angles, and dihedral angles) of NIC and NIC–OXA salt along with their respective experimental values (Jarzembska et al., 2014; Shimpi et al., 2017) are given in Supplementary Table S1. A comparison with the experimental/theoretical geometrical parameters of NIC and NIC–OXA salt revealed that the values are the same, which are within 0.017/0.003 Å, 0.5°/0.7°, and 0.5°/1.7° in bond lengths, bond angles, and dihedral-angles, respectively, except for the bonds having one hydrogen atom. The considerable difference of 0.005 Å is noticed in the calculated bond lengths values of (C18–N7) and (C34–N23) bonds of NIC than NIC–OXA salt, confirming the presence of intermolecular hydrogen bonds (N7–H8⋯O1═C3) and (N23–H24⋯O4═C6). Corresponding changes are observed in the bond angles and dihedral angles of these bonds.

In the same manner, after comparing the geometrical parameters of OXA with NIC–OXA salt, it is observed that the values do not differ by more than 0.0045/0.013 Å in bond lengths, 2.9°/3.5° in bond angles, and 0.0°/0.0° in dihedral angle, excluding the bonds accompanying a hydrogen atom. A difference of 0.0223 Å has been found between the observed and simulated bond-length values of (C6–O5) and (C3–O2) bonds of OXA from NIC–OXA salt. It seems that in OXA, a hydrogen atom is attached to O5 and O1 atoms, whereas these are transferred to pyridine N7 and N23 atoms of NIC resulting in (N7–H8) and (N23–H24) bonds. The corresponding changes are noticed among bond angles and dihedral angles of aforesaid bonds, confirming the presence of strong intermolecular hydrogen bonding (N7–H8⋯O5–C6) and (N23–H24⋯O2–C3). The calculated bond-length values (1.2082 Å) of (C3═O1) and (C6═O4) groups in NIC–OXA salt are slightly larger than the value of OXA (1.1988 Å), due to the fact that this bond is free in OXA, while it is involved in intermolecular hydrogen bonding in NIC–OXA salt.

The optimized geometrical parameters of NIC–OXA salt are also compared with their respective experimental values (Shimpi et al., 2017) (as given in Supplementary Table S1). It is clear from Supplementary Table S1 that the calculations can replicate the experimental values of bond length, bond angle, and dihedral angle of 0.050 Å, 1.3°, and 4.3°, respectively, except for bonds with one of the atoms as hydrogen. However, the noticeable differences of (0.149/0.147 Å) among simulated and experimental bond-length values of (N13H14/N13H15) and (N29H30/N29H31) of NIC are present because these groups are involved in intermolecular hydrogen bonding with carbonyl (C═O) groups of neighboring OXA/NIC molecules in NIC–OXA salt (as shown in Supplementary Figure S7). The consequent changes are also found among bond-angle and dihedral-angle values of respective bonds. Likewise, in (N7–H8) and (N23–H24) bonds, the differences in bond-length values of 0.893 Å are found because in optimized geometry (Figure 1), the intermolecular hydrogen bonds (O5⋯H8) and (O2⋯H24) are stronger than the crystal structure (Shimpi et al., 2017), as given in Supplementary Table S1, except that these minor changes are noticed among experimental and calculated bond-length values of 0.100/0.100, 0.088/0.088, and 0.064/0.064 Å in (C19–H20)/(C35–H36), (H17–C18)/(H33–C34), and (C10–H22)/(C26–H38) bonds, respectively. The comparative investigation of structural parameters between experimental and theoretical values reveals that both match well. However, some differences arise due to the fact that calculations were performed for isolated molecules, whereas the experiment was done for the bulk state where intermolecular interactions were already taken into account.

The minimized ground state energies of NIC, OXA, and NIC–OXA salt are −261,740.63, −237,476.74, and −760,970.00 kcal mol^−1^, respectively. The binding energy (BE) of NIC–OXA salt is calculated as the difference between the energy of NIC–OXA salt and the sum of its two constituents; NIC and OXA and are computed as −12 kcal mol^−1^. Due to the basis set superposition error, the computed BE of NIC–OXA salt is corrected by counterpoise correction (Runge and Gross, 1984) and is calculated to be −10.55 kcal mol^−1^.

### 4.2 Spectroscopic (IR and Raman) Assignments

**FIGURE 2:**
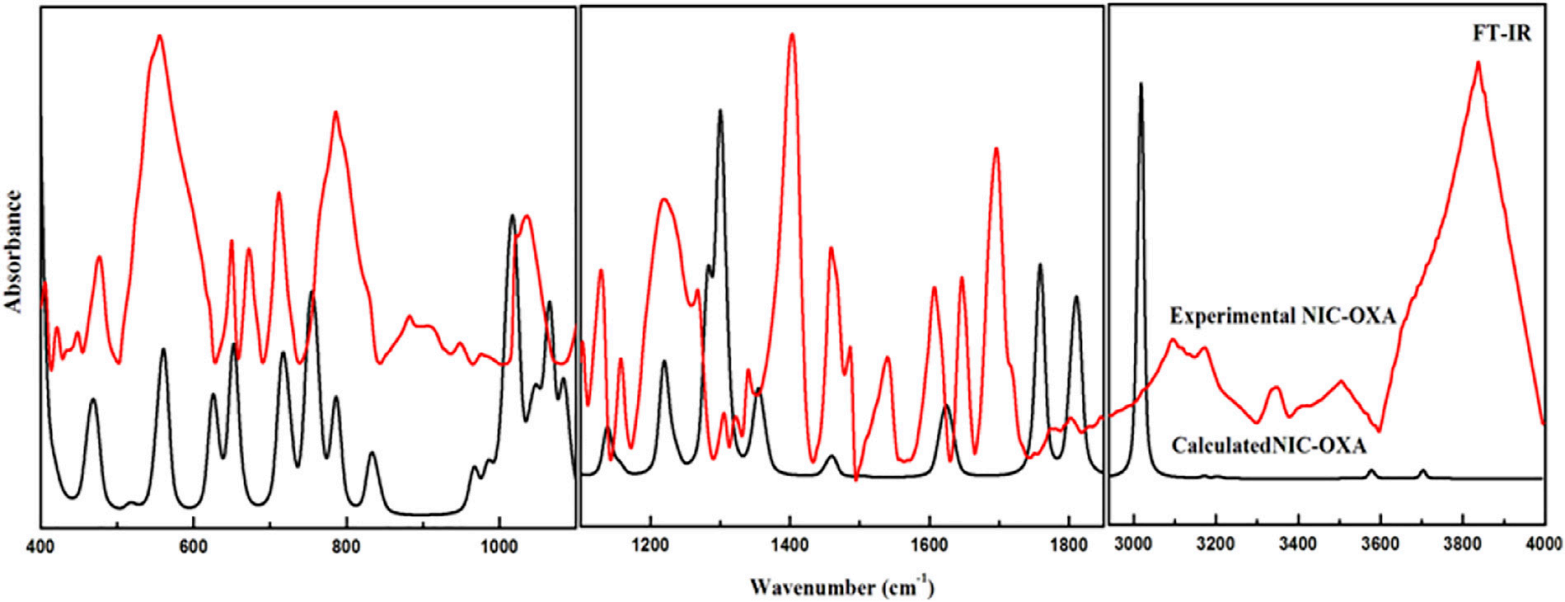
Experimental and calculated FT-IR absorbance spectra of NIC–OXA salt in the region of 400–4,000 cm^−1^. FT-IR, Fourier transform IR; NIC–OXA, nicotinamide–oxalic acid.

**FIGURE 3:**
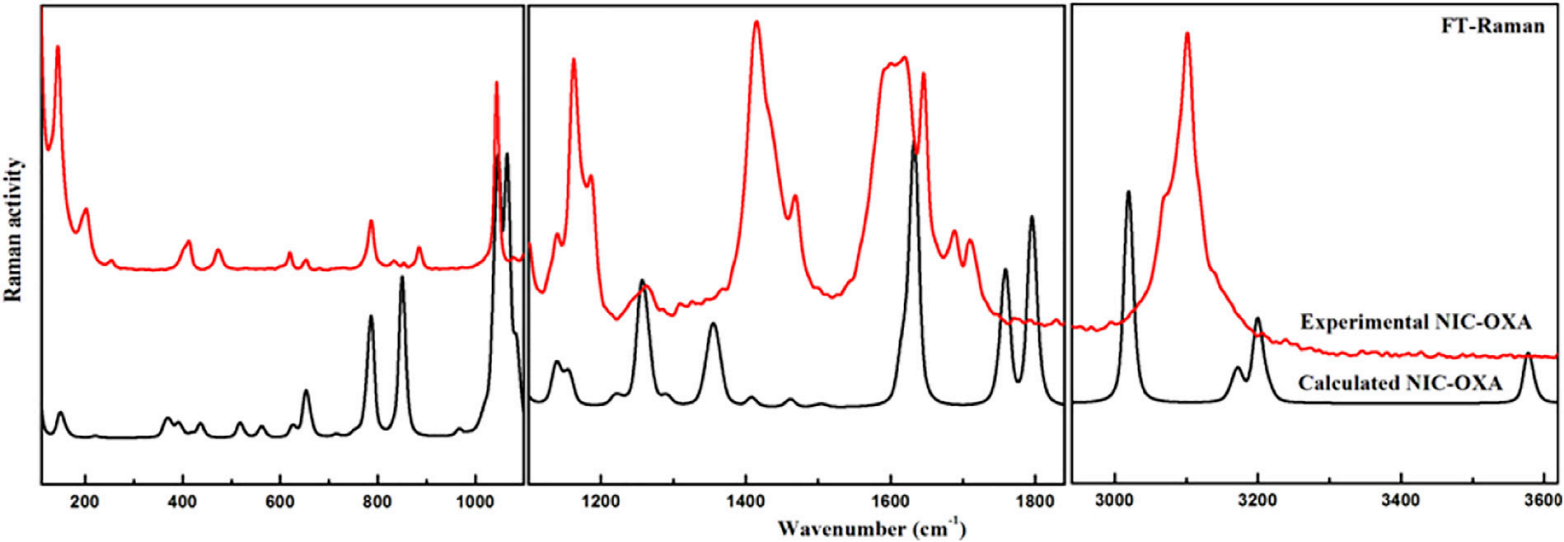
Experimental and calculated FT–Raman spectra of NIC–OXA salt in the region of 400–3,620 cm^−1^. FT, Fourier transform; NIC–OXA, nicotinamide–oxalic acid.

The number of atoms in NIC, OXA, and NIC–OXA salt is 15, 8, and 38, which give rise to 39, 18, and 108 (3N-6) normal modes of vibrations, respectively. The simulated vibrational wavenumbers along with their PED assignments for NIC, OXA, and NIC–OXA salt are given in Supplementary Tables S2–S4, respectively. A comparison is made between observed and calculated IR and Raman spectra of NIC and OXA as shown in Supplementary Figures S81–S11, respectively. Further, the comparison of observed and simulated bond lengths with stretching wavenumbers of groups involved in hydrogen bonding and IR/Raman spectra of NIC–OXA salt is given in Table 1 and Figures 2, 3, respectively, as discussed below.

**TABLE 1:** Observed and calculated bond length and stretching frequency of modes involved in hydrogen bonding.

Molecule	C–N of pyridine ring of NIC			C═O group of OXA			C–H of pyridine ring of NIC		
	Bond length (Å)	Stretching frequency		Bond length (Å)	Stretching frequency		Bond length (Å)	Stretching frequency	
	—	IR	Raman	—	IR	Raman	—	IR	Raman
Experimental									
NIC	1.3408	1,264	1,276	—	—	—	1.0830	3,013 (C6–H12)	3,009 (C6–H12)
OXA	—	—	—	1.2100	1,689 (C2═O3)	1,691 (C2═O3)	—	—	—
	—	—	—	1.2100	1,689 (C6═O7)	1,691 (C6═O7)	—	—	—
NIC–OXA salt	1.3446	1,267	1,260	1.2150	1,695 (C3═O1)	1,689 (C3═O1)	1.0190	3,095 (C10–H22)	3,102 (C10–H22)
	1.3446	1,267	1,260	1.2150	1,695 (C6═O4)	1,689 (C6═O4)	1.0190	3,095 (C26–H38)	3,102 (C26–H38)
Theoretical									
NIC	1.3350 (C6–N3)	1,272	1,272	—	—	—	1.0868	3,010 (C6–H12)	3,010 (C6–H12)
OXA	—	—	—	1.1988	1788 (C2═O3)	1788 (C2═O3)	—	—	—
	—	—	—	1.1988	1788 (C6═O7)	1788 (C6═O7)	—	—	—
NIC–OXA salt	1.3397 (C18–N7)	1,268	1,268	1.2082	1759 (C3═O1)	1759 (C3═O1)	1.0832	3,071 (C10–H22)	3,071 (C10–H22)
	1.3397 (C34–N23)	1,268	1,268	1.2082	1759 (C6═O4)	1759 (C6═O4)	1.0832	3,071 (C26–H38)	3,071 (C26–H38)

Note. NIC, nicotinamide; OXA, oxalic acid.

#### 4.2.1 Pyridine Rings (R1 and R2) Vibrations

The C–C stretching vibrations of NIC and NIC–OXA salt mixed with C-N stretching and C–H in-plane bending and asymmetric deformation of the ring were calculated at 1,578 and 1,603 cm^−1^ and are in good agreement with the observed bands at 1,575/1,579 and 1,607/1,600 cm^−1^, respectively, in IR/Raman spectra. Asymmetric deformation of the ring in NIC and NIC–OXA salt calculated at 619 and 652 cm^−1^ was assigned to the peak at 620/629 and 650/651 cm^−1^, respectively, in IR/Raman spectra. Asymmetric torsion of the ring is calculated at 418 and 419 cm^−1^ in NIC and NIC–OXA salt and assigned to the peak at 411/415 and 420/412 cm^−1^, respectively, in IR/Raman spectra.

#### 4.2.2 Carboxamide Group Stretching Vibrations

NIC molecule consists of a carboxamide group, attached to the meta-3 position. The carbonyl (C═O) group stretching vibrations usually lie in the region of 1,700–1,800 cm^−1^ (Socrates, 1980). The C═O stretching mode of NIC (C4═O1) and NIC–OXA salt (C11═O9 and C27═O25) is found at 1,712 and 1,722 cm^−1^ and can be observed at 1,674/1,677 and 1,647/1,645 cm^−1^ in IR/Raman spectra, as depicted in Supplementary Figure S3 and Figure 1, respectively. In both of the molecules, this group was observed at the lower wavenumber region due to its involvement in intermolecular hydrogen bonding in the crystalline state as shown in Supplementary Figures S5, S7, respectively.

The N–H stretching vibrations of the amide (NH_2_) group are dependent on the strength of hydrogen bonding (Cao et al., 2017). The N2–H10 and N2–H11 stretching vibration modes of NIC (Supplementary Figure S3) are calculated at 3,523 and 3,410 cm^−1^, respectively, which correspond to the observed peak at 3,360/3,372 cm^−1^ in IR/Raman spectra. The decrease in wavenumber is due to the involvement of these groups in intermolecular hydrogen bonding (N2–H10⋯O═C) and (N2–H11⋯N–C) in the crystal structure of NIC (Supplementary Figure S5). However, the stretching vibration of (N2–H10) is calculated at a higher wavenumber than that of (N2–H11), which is because the bond length of the former (1.0063 Å) is less than that of the latter one (1.0087 Å), as shown in Supplementary Figure S3.

Similarly, in NIC–OXA salt, the stretching modes of (N13–H15)/(N29–H31) and (N13–H14)/(N29–H30) are respectively calculated at 3,511/3,511 and 3,401/3,401 cm^−1^, corresponding to the observed peak at 3,379/3,360 cm^−1^ in the IR/Raman spectra region. Again this decrease in wavenumber is due to the involvement of these groups in intermolecular hydrogen bonding (N13–H15/N29–H31⋯O═C) and (N13–H14/N29–H30⋯O═C) with neighboring OXA and NIC molecules, respectively, in the crystalline state (Supplementary Figure S7). The higher value of calculated stretching vibrations of (N13–H15)/(N29–H31) than (N13–H14)/(N29–H30) is due to the decrement in the bond length of the previous bond (1.0072 Å) than the next one (1.0095 Å), as given in Figure 1.

#### 4.2.3 Modes Involved in Intermolecular Hydrogen Bonding

In Figure 1, the C═O and CO of OXA are involved in intermolecular hydrogen bonding with CH/N–H and N–H groups of NIC molecule, respectively (as the hydrogen from COOH of OXA was transferred toward the N atom of the pyridine ring in NIC attributed to the formation of NIC–OXA salt). The free N–H stretching vibrations are found in the region of 3,400–3,600 cm^−1^, while the presence of intermolecular hydrogen bond can shift this towards lower wavenumber values (Wong, 1996). It is clear that in NIC–OXA salt, the (N23–H24) and (N7–H8) stretching vibrations are observed in a lower wavenumber (2,852 cm^−1^ in the IR spectrum) than calculated values (2,897 and 2,894 cm^−1^, respectively), which confirms the presence of intermolecular hydrogen bonding (N23–H24⋯O2) and (N7–H8⋯O5). However, in NIC, no such peak was present (Supplementary Figure S3 and Supplementary Table S2).

The C═O group stretching vibrations usually lie in the region of 1,680–1780 cm^−1^ (Shreve et al., 1951). The stretching vibration modes (C3═O1) and (C6═O4) in NIC–OXA salt are calculated at 1,759 cm^−1^, corresponding to the observed peaks at 1,695/1,689 cm^−1^ in IR/Raman spectra (Table 1). This downward shifting in the observed value confirms the presence of intermolecular hydrogen bonding (C10–H22⋯O1═C3), (N7–H8⋯O1═C3), (C26–H38⋯O4═C6), and (N23–H24⋯O4═C6) in the crystal structure, as shown in Figure 1 and Supplementary Figure S7. The carbonyl (C2═O3) and (C6═O7) groups’ stretching vibrations of individual OXA are found at 1,788 cm^−1^ and assigned to the peaks at 1,689/1,691 cm^−1^ in IR/Raman spectra (Supplementary Figure S4). Again, this decrease in wavenumber is because they are forming an intermolecular hydrogen bond with hydroxyl (OH) groups of the neighboring molecules (Supplementary Figure S6).

The (C10–H22)/(C26–H38) mode stretching vibrations of NIC–OXA salt are calculated at 3,071 cm^−1^, corresponding to observed peaks at 3,095/3,102 cm^−1^ in IR/Raman spectra. The little increment in wavenumber is because of weak intermolecular hydrogen bonds (C10–H22⋯O1═C3) and (C26–H38⋯O4═C6), which are also attributed to the same decrement in observed bond lengths (1.0190 Å) of both the C–H bonds with a simulated value (1.0832 Å) (as shown in Figure 1 and Supplementary Figure S7).

Hence, from the above discussions, it can be said that the functional groups are free and hydrogen bonded in NIC, OXA, and NIC–OXA salt.

### 4.3 X-Ray Powder Diffraction Pattern

The XRPD pattern is characterized by a unique combination of position and intensity of Bragg peaks, which determine or are used to evaluate every crystalline phase present in the sample. Thus, every individual crystalline material has its own “fingerprint” that helps in the utilization of XRPD data in phase identification. The best way to identify the bulk purity of crystalline samples using powder diffraction data requires a comparison with the simulated diffraction pattern from the crystal structure. Powder diffraction data for the obtained product NIC–OXA salt sample and diffraction pattern generated using a single crystal structure are compared in Supplementary Figure S1. The single crystal structure for NIC–OXA salt was reported in 2017 (Shimpi et al., 2017). The analysis of powder patterns indicates the presence of a single crystalline phase in the bulk sample.

### 4.4 Differential Scanning Calorimetry Analysis

DSC is often used to identify solid-state forms (starting compound, polymorph, etc.) and assess their thermal stability. DSC analysis validates an endothermic transition peak at 178.7°C during the heating in DSC, which further corresponds to the melting phenomenon in crystalline NIC–OXA salt sample (as shown in Supplementary Figure S2), and the transition is in line with the reported data (Shimpi et al., 2017).

### 4.5 Topological and Energy Parameters at Bond Critical Points

QTAIM is an important tool for studying non-covalent hydrogen bonding interactions, which are relevant in supramolecular chemistry, atmospheric chemistry, finding the structure of proteins, and crystal engineering (Bohórquez et al., 2011). The QTAIM successfully defines the criteria for the existence of hydrogen bonds based on BCPs and paths of electron density (ED). The hydrogen bond exists if the following criteria are followed: ED (ρ_BCP_) and Laplacian of ED (∇^2^ρ_BCP_) are within the range of 0.002–0.040 a.u. and 0.024–0.139 a.u., respectively, which predict the structural stability (Koch and Popelier, 1995).

Conventional bonding and unconventional hydrogen bonding are associated with strong and weak bonds, respectively. The unconventional (C–H⋯O) interaction is important in designing and formulating drugs (Desiraju, 1995). The relationship between hydrogen bond energy (E) and potential energy density (V_BCP_) is written as E = 0.5 V_BCP_ (Espinosa et al., 1998). The total energy density (H_BCP_) is the sum of kinetic electron energy density (G_BCP_) and V_BCP_, as follows:

$$H_{BCP}\;=\;\left(G_{BCP}\;+\;V_{BCP}\right)$$

**FIGURE 4:**
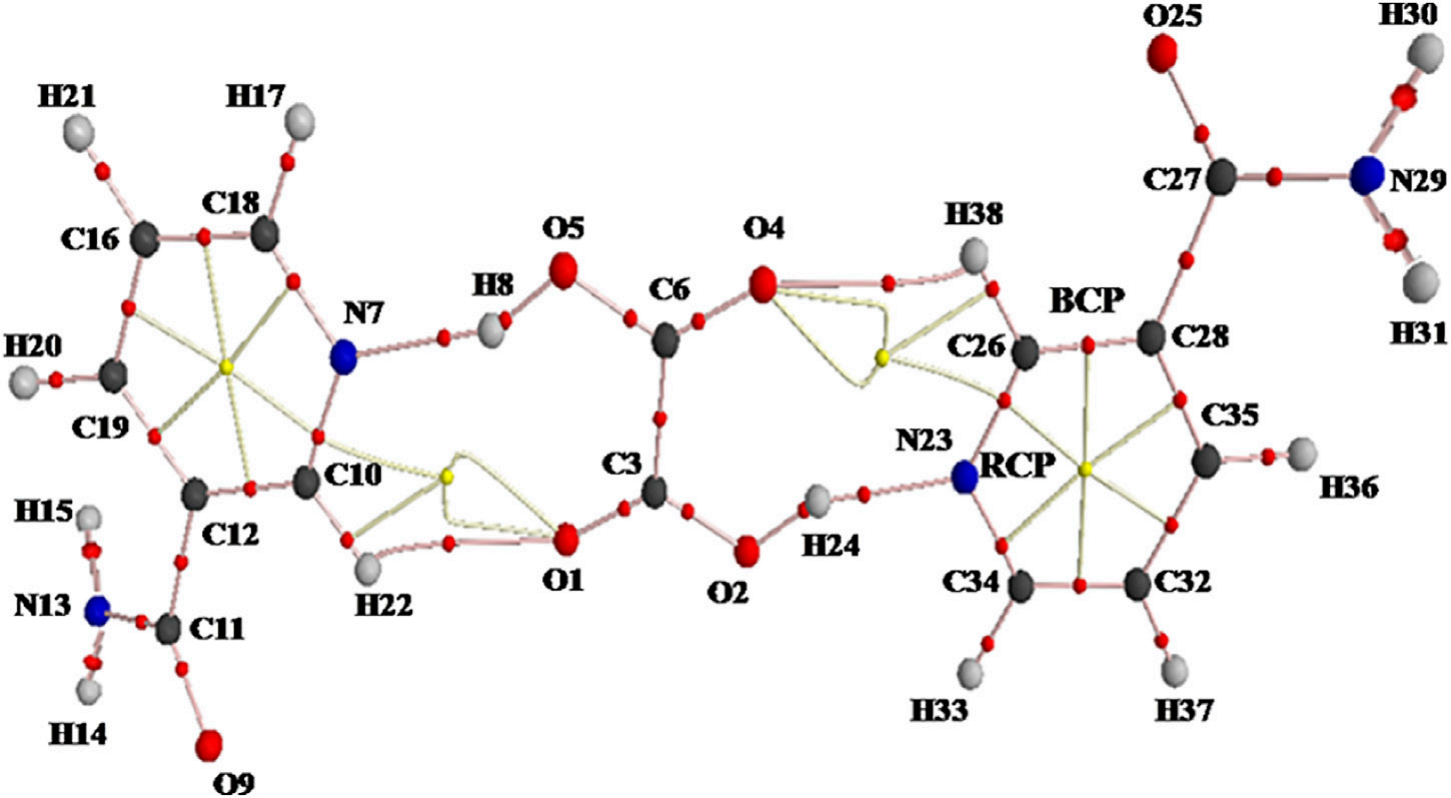
AIM molecular graph showing ring critical points (RCPs; yellow small balls), bond critical points (BCPs; red small balls), and bond paths (pink lines) of NIC–OXA salt calculated with B3LYP/6-311++G(d, p) level. AIM, atoms in molecules; NIC–OXA, nicotinamide–oxalic acid.

The molecular graph and geometrical parameters for the existence of hydrogen bonds of NIC–OXA salt are shown in Figure 4 and Supplementary Table S4, respectively. Topological parameters and energy of interaction for intermolecular hydrogen bonds of NIC–OXA salt are listed in Table 2.

**TABLE 2:** Geometrical parameter (bond length) and topological parameters for bonds of interacting atoms of intermolecular hydrogen bonding of NIC–OXA salt: electron density (ρ_BCP_), Laplacian of electron density (∇^2^ρ_BCP_), electron kinetic energy density (G_BCP_), electron potential energy density (V_BCP_), total electron energy density (H_BCP_) at bond critical point (BCP), and estimated interaction energy (E_int_).

Hydrogen bonds	Bond length (Å)	ρ_BCP_ (a.u.)	∇^2^ρ_BCP_ (a.u.)	G_BCP_ (a.u.)	V_BCP_ (a.u.)	H_BCP_ (a.u.)	E_int_ (kcal mol^−1^)	G_BCP_/ρ_BCP_
N7–H8⋯O5	1.0077	0.0471	0.1015	0.0077	−0.0407	−0.0330	−12.7698	0.1635
N23–H24⋯O2	1.0077	0.0471	0.1015	0.0077	−0.0407	−0.0330	−12.7698	0.1635
C10–H22⋯O1	2.4165	0.0097	0.0378	−0.0016	−0.0062	−0.0078	−1.9453	−0.1649
C26–H38⋯O4	2.4165	0.0097	0.0378	−0.0016	−0.0062	−0.0078	−1.9453	−0.1649

NIC–OXA salt is the result of an acid–base reaction or neutralization reaction. In this reaction, the proton donor (OXA) and proton acceptor (NIC) groups interact with each other to form a single crystal phase, salt (cation and anion). This interaction is due to strong intermolecular hydrogen bonds to hold them together and is directly responsible or is the key driving force for crystal formation. Strong hydrogen bonds are especially predominant between acids (A) and their conjugate bases (B), B–H⋯A^−^ (Hibbert and Emsley, 1990)**.** Since NIC–OXA salt possesses the protonated base, N^+^–H⋯O^−^, it has a tendency to form strong hydrogen bonds.

In NIC–OXA salt, the intermolecular hydrogen bonds H8⋯O5, H24⋯O2, H22⋯O1, and H38⋯O4 are medium and partially covalent due to (∇^2^ρ_BCP_) > 0 and H_BCP_ < 0 as given by Rozas et al. (Rozas et al., 2000), whereas (N7–H8⋯O5) and (N23–H24⋯O2) are strong hydrogen bonds having the value of ratio G_BCP_/ρ_BCP_ of less than 1 (0.1635) (Gilli and Gilli, 2009; Low et al., 2019). Further, as per the Desiraju criteria (Desiraju, 1995), (N7–H8⋯O5) and (N23–H24⋯O2) are conventional hydrogen bonds with the same higher interaction energy (−12.7698 kcal mol^−1^), while (C10–H22⋯O1) and (C26–H38⋯O4) are unconventional hydrogen bonds in NIC–OXA salt (Table 2).

### 4.6 Natural Bond Orbital Analysis

NBO is the most promising approach to chemically interpret the conjugative or hyper conjugative and delocalized charge transfer interactions from the filled lone pair donor to an empty acceptor (Pir et al., 2012). The determination of the stabilization energy (E^2^) that is associated with delocalized electron between the filled donor (i) and empty acceptor (j) NBO is given as follows:

E^2^ = $\;-n_{\sigma }\left[\frac{<\sigma \left|F\right|\sigma {>}^{2}}{\varepsilon_{\sigma^{*}}-\varepsilon_{\sigma }}\right]$ = $-n_{\sigma }\left[\frac{F_{\mathrm{ij}}^{2}}{\Delta E}\right]$ where n_σ_ refers to the population of donor orbital $<\text{σ}\left|\text{F}\right|\text{σ}{>}^{2}$ , or ${\text{F}}_{\text{ij}}^{2}$ is the Fock matrix element between i and j NBO orbitals, and ε_σ_* and ε_σ_ are the energies of σ* and σ NBOs (Pir et al., 2012).

This approach confirms the presence and strength of intra- and intermolecular hydrogen bond interactions arising from E^(2)^ and hence reflects the structural features of molecules (Nagy, 2014). The values of E^(2)^ with the corresponding intermolecular hydrogen bonding interactions between donor and acceptor orbitals of NIC–OXA salt are listed in Supplementary Table S5. It is clear from Supplementary Table S5 that the two strong intramolecular hydrogen bond interactions—[LP (O2) →π*(O1═C3)] and [LP (O5) → π*(O4═C6)]—within OXA have been observed to have stabilization energy of 56.78 kcal mol^−1^. These interactions are responsible for a decrease in orbital occupancy of these lone pairs (1.76729) in comparison to other bonds. Similarly, in NIC molecules, interactions are observed within (unit 2) and (unit 3) in amide (N13H_2_ and N29H_2_) and carbonyl (C11═O9 and O25═C27), i.e., [LP(1)N13 → π*(O9═C11)] (NIC1) and [LP(1)N29→ π*(O25═C27)] (NIC2) with same stabilization energy of 39.96 kcal mol^−1^, providing more stability to NIC–OXA salt.

The intermolecular hydrogen bond interactions between LP(1)O1 (OXA), LP(1)O4 (OXA), LP(1)O5 (OXA), and LP(1)O2 (OXA) and are σ*(C10–H22) (NIC1), σ*(C26–H38) (NIC2), σ*(N7–H8) (NIC1), and σ*(N23–H24) (NIC2), respectively, confirming the presence of strong hydrogen bonding with their strength (N7–H8⋯O5)/(N23–H24⋯O2) (27.39 kcal mol^−1^) > (C10–H22⋯O1)/(C26–H38⋯O4) (0.61 kcal mol^−1^), leading to the formation of NIC–OXA salt.

### 4.7 Frontier Molecular Orbital (Highest Occupied Molecular Orbital–Lowest Unoccupied Molecular Orbital) Analysis

**FIGURE 5:**
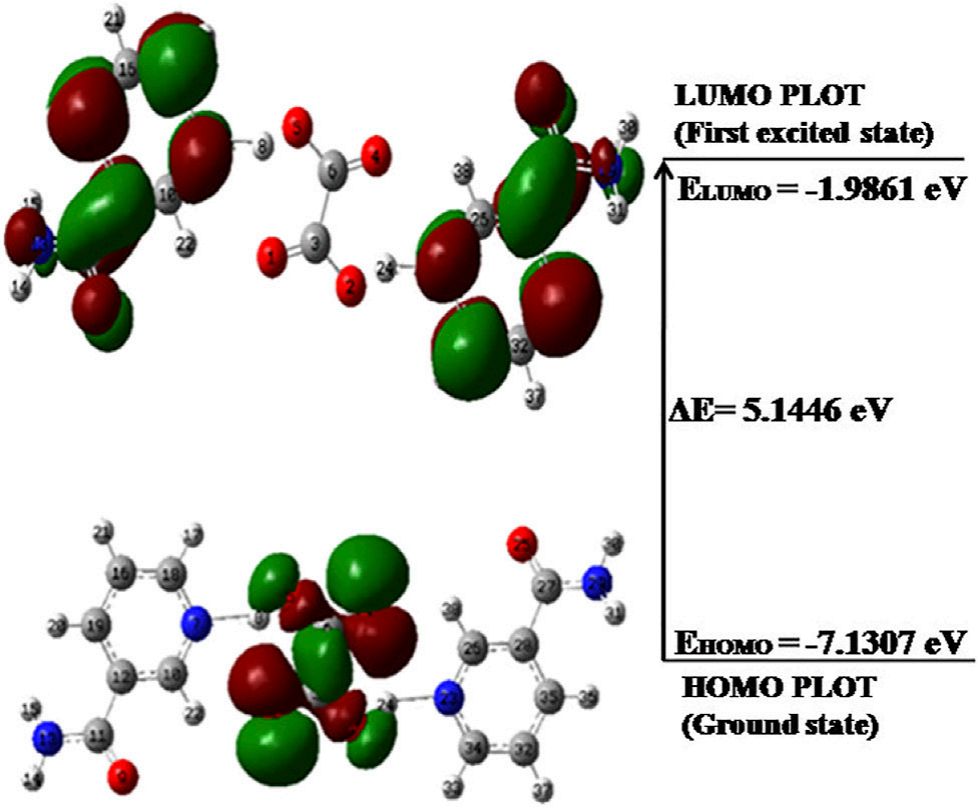
HOMO-LUMO energy gap of NIC–OXA salt with orbitals participating in electronic transitions. HOMO, highest occupied molecular orbital; LUMO, lowest unoccupied molecular orbital; NIC–OXA, nicotinamide–oxalic acid.

The FMO theory suggests that the interactions between FMOs (HOMO and LUMO) are due to the electronic transition states (Zhuo et al., 2012). These FMOs describe the electrical and optical properties of the molecules (Oyeneyin et al., 2018). The energy gap between HOMO and LUMO denotes the stability of the molecule. The large energy gap of a molecule means smaller electronic transition, less reactivity plus more stability, and vice versa (Khan et al., 2017). The HOMO-LUMO energy plots of NIC, OXA, and NIC–OXA salt are given in Supplementary Figures S11, S13, and Figure 5, respectively.

It is clear from Figure 5 that in NIC–OXA salt, the HOMO is localized on OXA; however, LUMO is distributed over both NIC molecules. It clearly demonstrates that the charge transfer takes place from OXA to NIC molecule in NIC–OXA salt. The HOMO-LUMO energy gaps of NIC, OXA, and NIC–OXA salt are 5.5356, 5.6667, and 5.1446 eV, respectively. It means that NIC–OXA salt represents higher electronic transitions as compared to NIC (API) and OXA (coformer). The previous work on NIC–CA cocrystals (Verma et al., 2019) reported that the energy gaps of CA and NIC–CA cocrystals were 7.2663 and 5.2331 eV, respectively. It means that the chemical reactivity lies in the order of NIC–OXA salt > NIC–CA cocrystal > NIC > OXA > CA.

Hence, it can be said that NIC–OXA salt is less stable and more reactive than NIC–CA cocrystal and NIC. It also validates how reactivity parameters are dependent on different coformers (OXA and CA) with common API (NIC), like in NIC–OXA salt and NIC–CA cocrystal.

### 4.8 Chemical Reactivity Descriptors

Chemical (global and local) reactivity descriptors help in the prediction of nucleophilic/electrophilic reagents as well as preferable attacking sites of any molecule (Frau and Glossman-Mitnik, 2018).

#### 4.8.1 Global Reactivity Descriptors

The calculated values of χ, S, η, ω, and μ for NIC, OXA, and NIC–OXA salt clearly define the trends in global reactivity parameters (Parr and Pearson, 1983; Geerlings et al., 2003; Chattaraj and Roy, 2007), as listed in Table 3. The high/low values of μ/χ and vice versa suggest that a molecule is nucleophilic and electrophilic in nature, respectively. The values of S and η of molecules can be described by their energy gaps. The molecules having large/small energy gaps are known as hard/soft molecules and typically are less/more polarizable. The high value of ω defines the tendency to attract more electrons from a donor molecule.

**TABLE 3:** Calculated E_HOMO_, E_LUMO_, energy band gap (E_L_–E_H_), chemical potential (μ), electro negativity (χ), global hardness (η), global softness (S), global electrophilicity index (ω), and maximum extent of charge transfer (ΔN_max_) at 298.15 K for nicotinamide (NIC), oxalic acid (OXA), and NIC–OXA salt using B3LYP/6-311++G(d,p).

Molecule	E_H_ (eV)	E_L_ (eV)	E_L_–E_H_ (eV)	χ (eV)	μ (eV)	η (eV)	S (eV)	ω (eV)	ΔN_max_
NIC	−7.3414	−1.8058	5.5356	4.5736	−4.5736	2.7678	0.1806	3.7788	1.6524
OXA	−8.1669	−2.5002	5.6667	5.3335	−5.3335	2.8333	0.1765	5.0200	1.8824
NIC–OXA salt	−7.1307	−1.9861	5.1446	4.5584	−4.5584	2.5723	0.1944	4.0390	1.7721

Note. NIC, nicotinamide; OXA, oxalic acid.

From Table 3, it is pointed out that μ for OXA (−5.3335 eV) < NIC (−4.5736 eV) and χ for OXA (5.3335 eV) > NIC (4.5736 eV). Hence, in NIC–OXA salt, OXA and NIC are of electrophilic and nucleophilic nature, respectively. The softness values lie in the order of NIC–OXA salt > NIC–CA cocrystal (Verma et al., 2019) > NIC > OXA > CA. The highest value of ω for OXA ascertains that it attracts more electrons from donor NIC molecules. ECT for OXA and NIC molecules was calculated to be 0.23, which shows that the transfer of charge occurs from API to coformer.

#### 4.8.2 Local Reactivity Descriptors

Fukui functions are the most prominent local reactivity descriptors that provide the details of electrophilic and nucleophilic sites selectivity (Bultinck et al., 2011). The local reactivity descriptors calculated for NIC–OXA salt are given in Supplementary Table S6. The maximum values of three descriptors for both O9 and O25 atoms recommended these sites for nucleophilic attack. The maximum values of ( $f_{k}^{-}$ , $s_{k}^{-},{\text{ω}}_{k}^{-}$ ) for C18 and C34 atoms suggest them to be demanding sites for electrophilic attack (Supplementary Table S6).

### 4.9 Molecular Electrostatic Potential Surface

**FIGURE 6:**
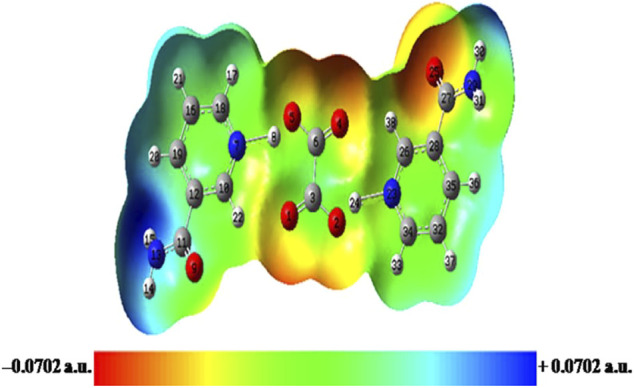
Molecular electrostatic potential (MESP) map of NIC–OXA salt formed by mapping of total electron density over electrostatic potential in the gas phase. NIC–OXA, nicotinamide–oxalic acid.

MESP map, the pictorial representation of electronic activity in a molecule, is drawn by plotting the electrostatic potential over an ED isosurface (Vijayalakshmi and Suresh, 2010). This map displays the molecular shape and size in terms of the positive (blue; electron-deficient), negative (red; electron-rich), and neutral (green) regions (Vijayalakshmi and Suresh, 2010) and further investigates the molecular structure by correlating the hydrogen-bonding interactions and physicochemical property relationship (Vidhya et al., 2019). In the MESP map, the red and blue colors denote the minimum and maximum electrostatic potential regions, which indicate the electron-donating and electron-accepting abilities for a given molecule, respectively. The MESP maps of NIC, OXA, and NIC–OXA salt are displayed in Supplementary Figures S14, S15, and Figure 6, respectively.

From Supplementary Figure S14, it is shown that C–N/C═O and C–H of the pyridine ring/NH_2_ group are in the red (suitable for electrophilic attack) and blue (prone to nucleophilic attack) regions, respectively. The MESP of OXA represents both the carbonyl (C═O) and hydroxyl (O–H) groups, which are appropriate for electrophilic and nucleophilic attacks, respectively (Supplementary Figure S15). Moreover, from the MESP of NIC–OXA salt, it is clear that electrophilic (N7–H8)/(N23–H24) plus (C6═O4)/(C3═O1) and nucleophilic (C10–H22)/(C26–H38) along with (C6–O5)/(C3–O2) groups, in NIC and OXA, becomes neutralized after the formation of NIC–OXA salt, clearly showing the presence of hydrogen bonding interactions (N7–H8⋯O5–C6), (N23–H24⋯O2–C3), (N7–H8⋯O1═C3), (N23–H24⋯O4═C6), (C10–H22⋯O1═C3), and (C26–H38⋯O4═C6) as shown in Figures 1, 6. Additionally, the amide (N13H_2_)/(N29H_2_) and carbonyl (C11═O9)/(C27═O25) groups are visualized as the electropositive (blue) and electronegative (red) regions, respectively, showing the highest tendency of participation in intermolecular hydrogen bonding in the crystal structure of NIC–OXA salt, as shown in Figure 6 and Supplementary Figure S7.

### 4.10 Molar Refractivity

MR is an important parameter in drug design and measures the size and total polarizability of a mole of compounds (Sawale et al., 2016). Polarizability depicts an important role in biological activities and molecular modeling (Hansch et al., 2003). Refractive index and electronic polarizability studies are useful for understanding the presence of interactions between molecules (Pacak et al., 2006). Both the MR and refractive index are factors that simultaneously increase with change in drug concentration, which means that packing of molecules becomes tighter due to interaction with an additional substance (Schneider, 20132000; Sliwoski et al., 2014). Lipinski’s rule of five validates drug-like or activeness properties of biological or pharmacological molecules, which may be used as an orally active drug in humans. To study the drug-likeness behavior of NIC–OXA salt, the aforesaid rule has been used. This rule states that the compound should possess 1) an MR of 40–130 e.s.u., 2) a molecular weight of 180–500 g/mol, and 3) a number of atoms between 20 and 70 (Lipinski et al., 1997).

The calculated MR values for NIC and NIC–OXA salt are 19.43 and 39.78 e.s.u., respectively. It means that the interactions in drug (NIC) increase due to the presence of coformer, i.e., OXA. The molecular weights of NIC and NIC–OXA salt are 122.12 and 334.27 g/mol, respectively, with the number of atoms in NIC–OXA salt being 38. Thus, NIC–OXA salt follows the criteria of drug-likeness by Lipinski’s rule (Lipinski et al., 1997) and hence may be used for pharmaceutical formulation.

## 5 Conclusion

The present work is focused on studying the structure–reactivity–properties of NIC–OXA salt and its comparison with NIC based on spectroscopic signatures and quantum chemical calculations. Additionally, the charge (proton) transfer phenomenon from OXA (coformer) to NIC (API) is described, which is responsible for forming NIC–OXA salt. As a result, (N7–H8)/(N23–H24) and (C6–O5)/(C3–O2), (C3═O1)/(C6═O4) groups of NIC and OXA, respectively, were forming intermolecular hydrogen bonds (N7–H8⋯O5–C6), (N7–H8⋯O1═C3), (N23–H24⋯O2–C3), and (N23–H24⋯O4═C6) in NIC–OXA salt. The prepared NIC–OXA salt was identified and characterized by FT-IR, FT-Raman, XRPD, and DSC patterns. The spectroscopic result suggested that the decrease in wavenumber of observed peaks of (N7–H8), (N23–H24), and (C3═O1), (C6═O4) groups of NIC and OXA, respectively, confirms the presence of intermolecular hydrogen bonding of (N7–H8⋯O5–C6), (N7–H8⋯O1═C3), (N23–H24⋯O2–C3), (N23–H24⋯O4═C6), (C10–H22⋯O1═C3), and (C26–H38⋯O4═C6) in NIC–OXA salt. QTAIM suggested the presence of two hydrogen bond interactions (N7–H8⋯O5) and (N23–H24⋯O2) with a maximum value of interaction energy of −12.7698 kcal mol^−1^. NBO analysis also revealed the existence of intermolecular hydrogen bond (N7–H8⋯O5)/(N23–H24⋯O2) having maximum stabilization energy of 27.39 kcal mol^−1^. The lesser energy gap (E_L_–E_H_) in NIC–OXA salt from both NIC and NIC–CA cocrystal suggested that the former one was chemically more reactive (less stable) and softer than the last two systems. From local reactivity descriptors, the most reactive sites for nucleophilic and electrophilic attacks were located over O9/O25 and C18/C34 atoms, respectively. The MESP map suggested that the electro-negative and electro-positive groups of NIC and OXA involved in hydrogen bonding neutralize after NIC–OXA salt formation. Moreover, the NIC–OXA salt follows the criteria of drug-like property and may be considered an orally active form. These results highlight the reactivity–property relationship of NIC before and after combining with coformer (OXA), which leads to NIC–OXA salt formation and may be further used in comparing similar systems in pharmaceutical fields.

## Data Availability

The original contributions presented in the study are included in the article/Supplementary Material, further inquiries can be directed to the corresponding authors.
