# Supplementary material for: Experimental and Quantum Chemical Studies of Nicotinamide-Oxalic Acid Salt: Hydrogen Bonding, AIM and NBO Analysis
Source: Front Chem. 2022 Mar 15;10:855132. doi: 10.3389/fchem.2022.855132 (PMC8965448; doi:10.3389/fchem.2022.855132)
Supplement: Supplementary file 1 [file DataSheet1.pdf]

Supplementary Material

Experimental and quantum chemical studies of nicotinamide-oxalic acid salt: hydrogen bonding, AIM and NBO analysis

Priya Verma[1], Anubha Srivastava[1], Poonam Tandon[1*], Manishkumar R. Shimpi[2,3*]
[1*]Department of Physics, University of Lucknow, Lucknow, 226 007, India.
[2]Department of Materials and Environmental Chemistry, Stockholm University, Svante Arrhenius väg 16c, 10691 Stockholm, Sweden.
[3*]Chemistry of Interfaces, Luleå University of Technology, SE-971 87, Luleå, Sweden.

***Correspondence:**
Poonam Tandon
poonam_tandon@yahoo.co.uk

Manishkumar R. Shimpi

manishkumar.shimpi@mmk.su.se

1 Supplementary Figures and Tables

The simulated and recorded XRPD pattern of nicotinamide-oxalic acid (NIC-OXA) salt is given in Figure S1. The DSC plot of NIC-OXA salt is shown in Figure S2. The optimized ground state structures of NIC and OXA are shown in Figures S3 and S4, respectively. The NIC-OXA salt belongs to centrosymmetric orthorhombic Pbca system with space group and unit cell parameters a= 7.3230(17) Å, b= 12.506(2) Å, c= 15.749(5) Å, α=β=γ=90˚. The crystal structures of NIC, OXA and NIC-OXA salt are shown in Figures S5, S6 and S7, respectively. Experimental and calculated IR and Raman spectra of NIC and OXA are shown in Figures S8, S9, S10 and S11, respectively. HOMO and LUMO plots of NIC and OXA with their energy gap are shown in Figures S12 and S13, respectively. The molecular electrostatic potential (MESP) surface of NIC and OXA are given in Figures S14 and S15, respectively.
The experimental and calculated geometric parameters of NIC and NIC-OXA salt are given in Table S1. Theoretical and experimental vibrational wavenumbers of NIC and OXA are listed in Tables S2 and S3, respectively. Geometrical parameters for the existence of hydrogen bond interactions in NIC-OXA salt are given in Table S4. Second-order perturbation theory analyses of the Fock Matrix, in the NBO basis for intra- and intermolecular interactions in NIC-OXA salt are given in Tables S5. Reactivity descriptors as Fukui functions (f_k^+ , f_k^-), local softness (s_k^+ , s_k^-), local electrophilicity indices (ω_k^+ , ω_k^-) for NIC-OXA salt using Hirshfeld atomic charges are given in Tables S6.

1.1 Supplementary Figures

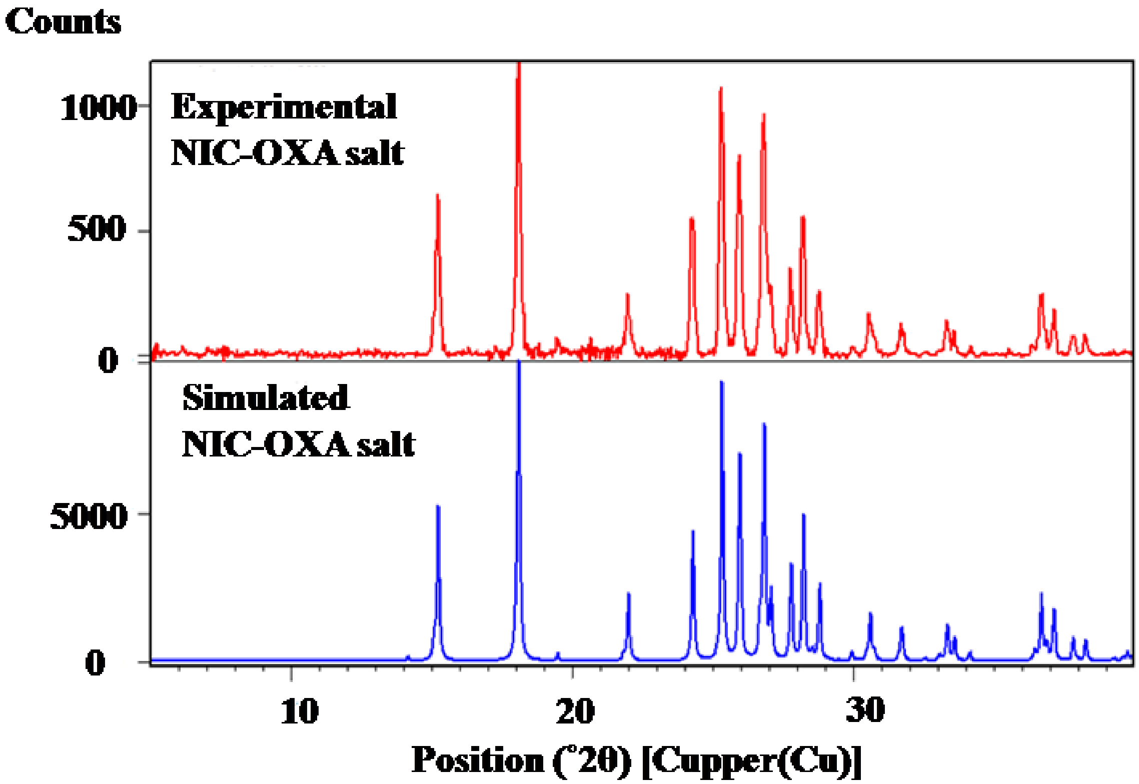

Figure S1. XRPD patterns of prepared NIC-OXA salt (top) and simulated pattern of NIC-OXA salt (bottom).

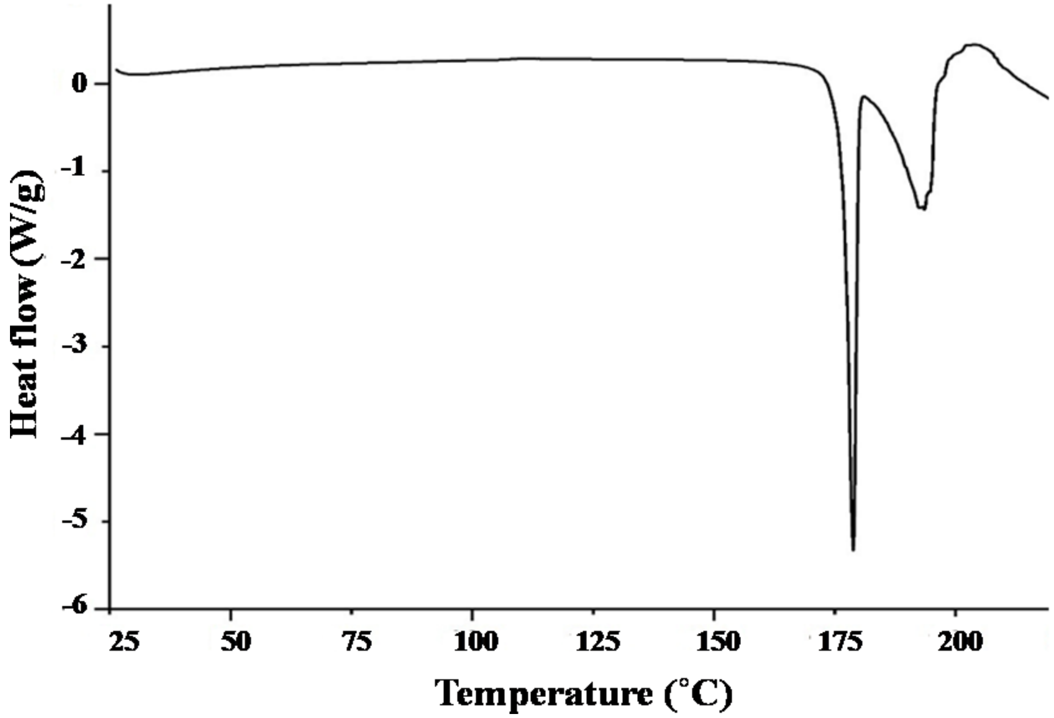

Figure S2. Differential scanning calorimetry (DSC) thermogram of NIC-OXA salt.

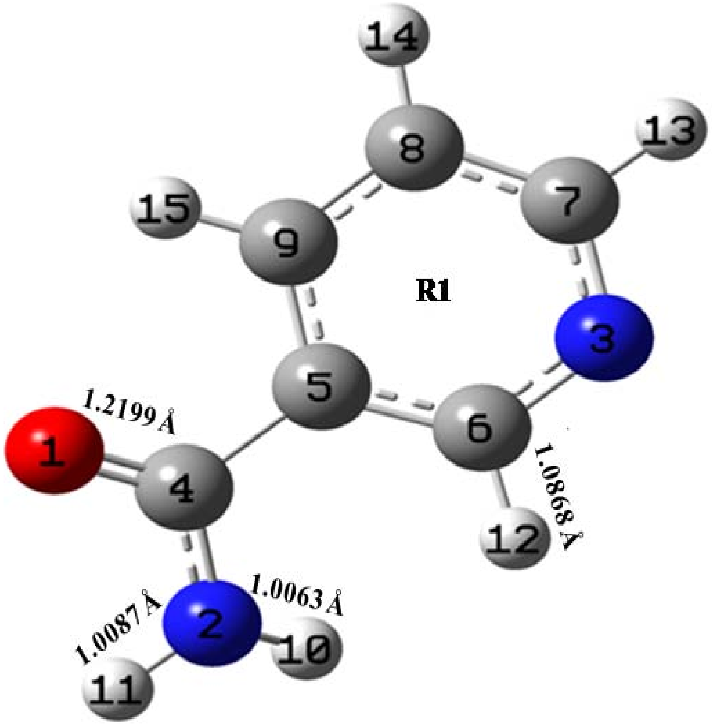

Figure S3. Optimized (ground state) structure of NIC with the atomic numbering used in this study.

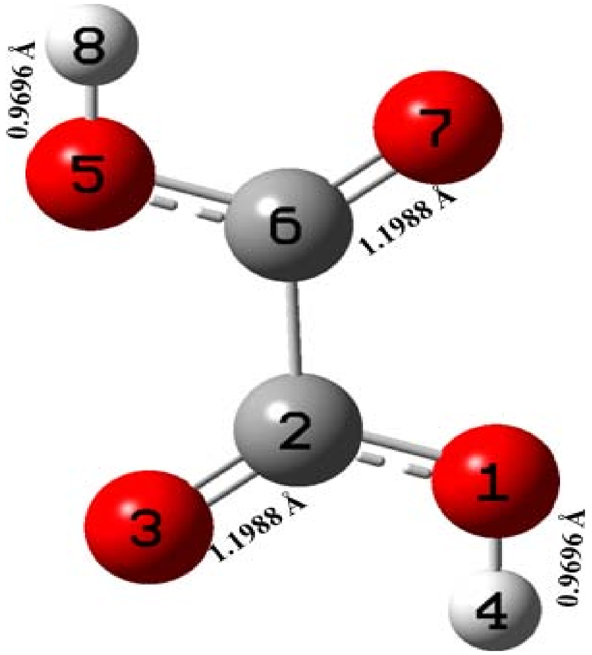

Figure S4. Optimized (ground state) structure of OXA with the atomic numbering used in this study.

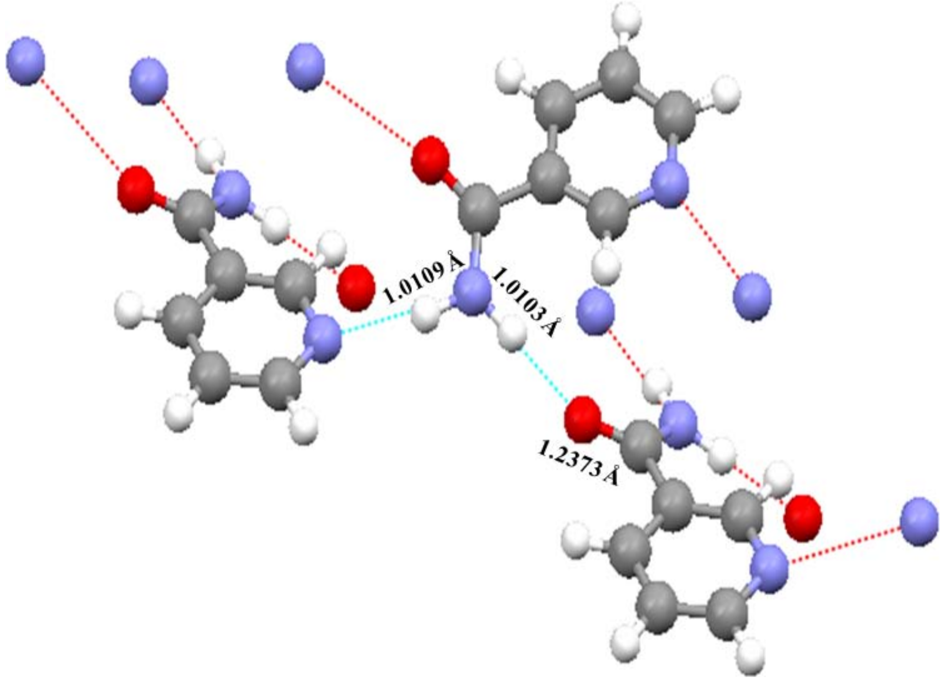

Figure S5. Crystallographic structure of NIC showing all the interactions.

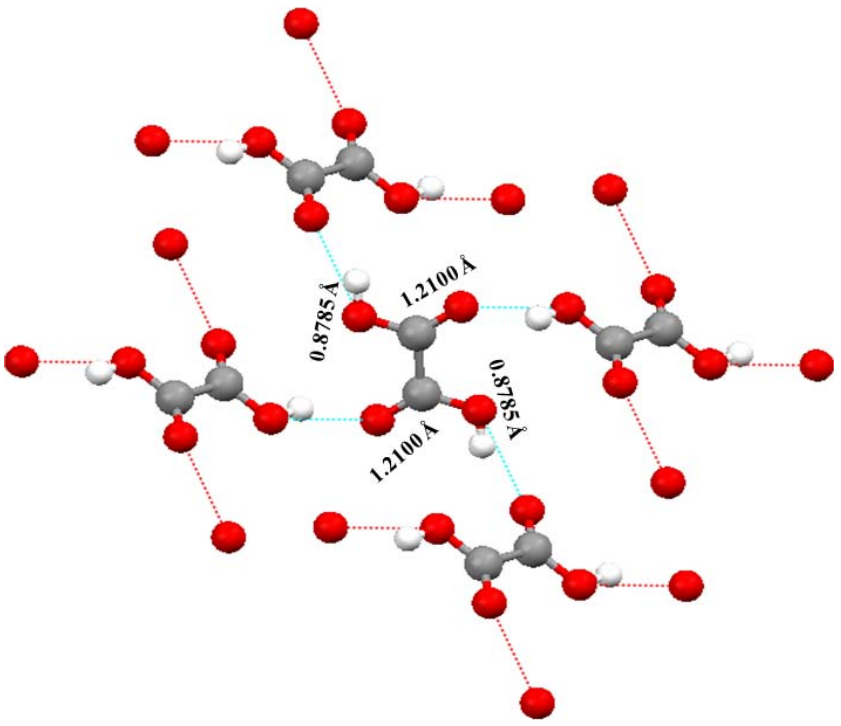

Figure S6. Crystallographic structure of OXA showing all the interactions.

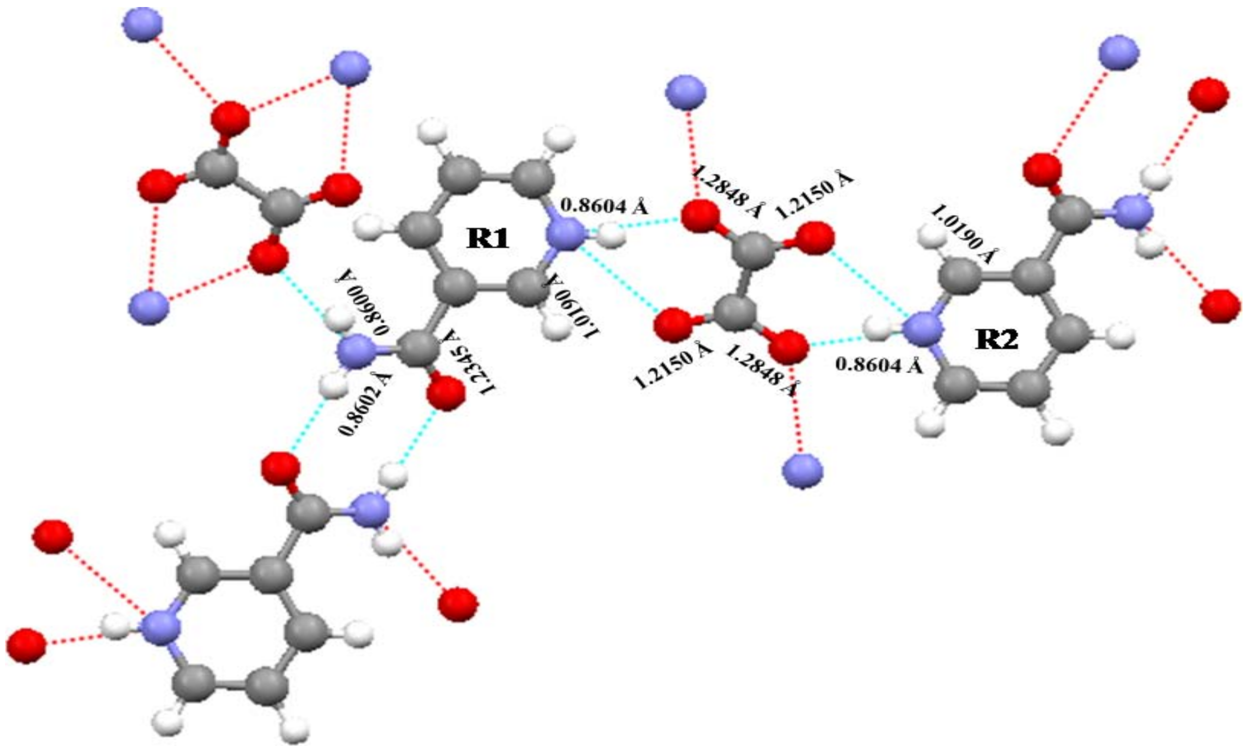

Figure S7. Crystallographic structure of NIC-OXA salt showing all the interactions.

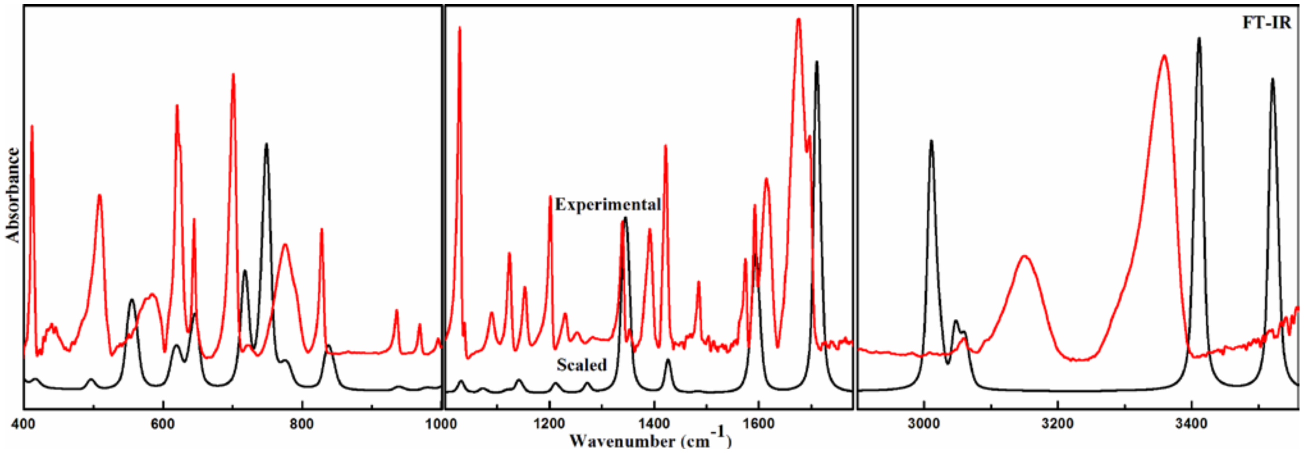

Figure S8. Experimental and calculated FT-IR spectra of NIC in the region of 400-2900 cm^{-1}.

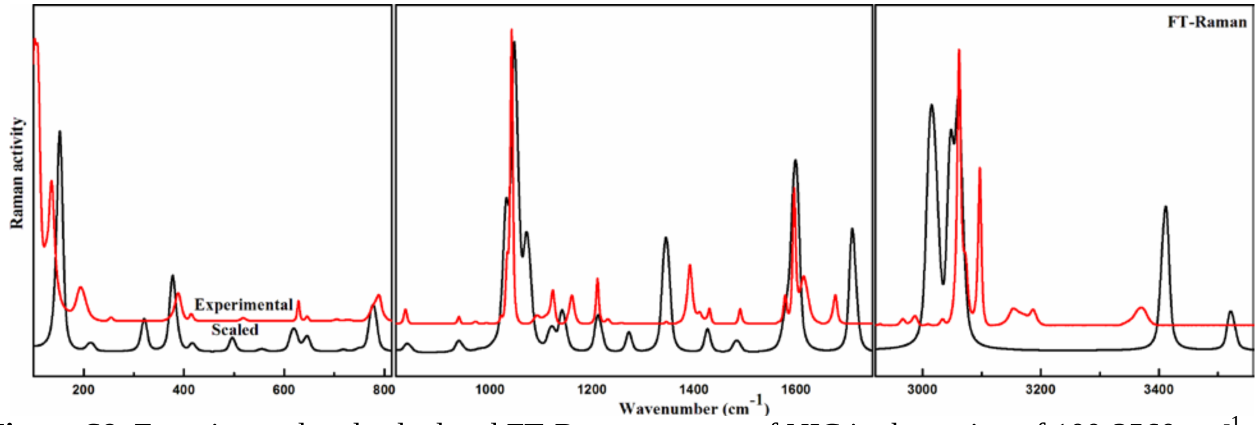

Figure S9. Experimental and calculated FT-Raman spectra of NIC in the region of 100-3560 cm^{-1}.

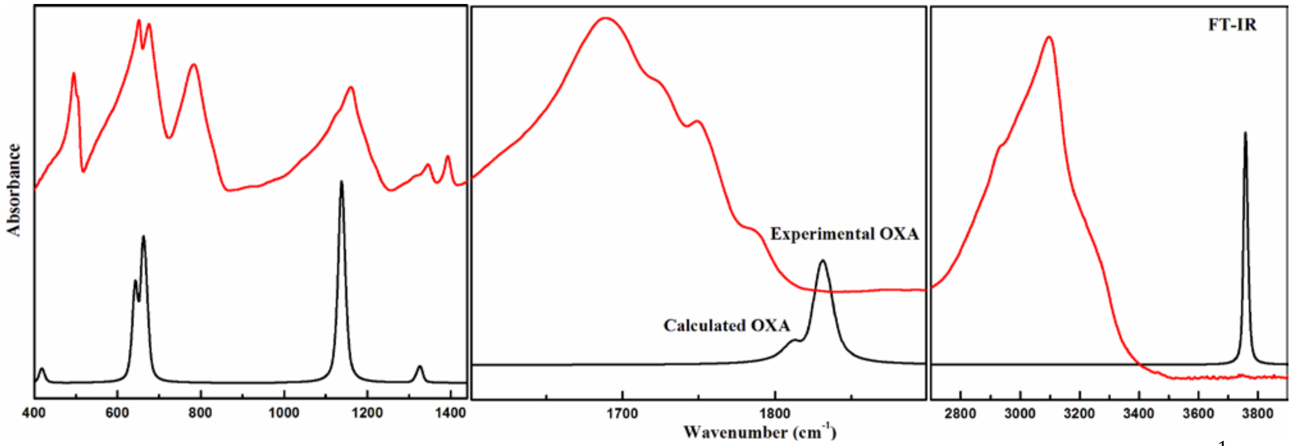

Figure S10. Experimental and calculated FT-IR spectra of OXA in the region of 400-3900 cm^{-1}.

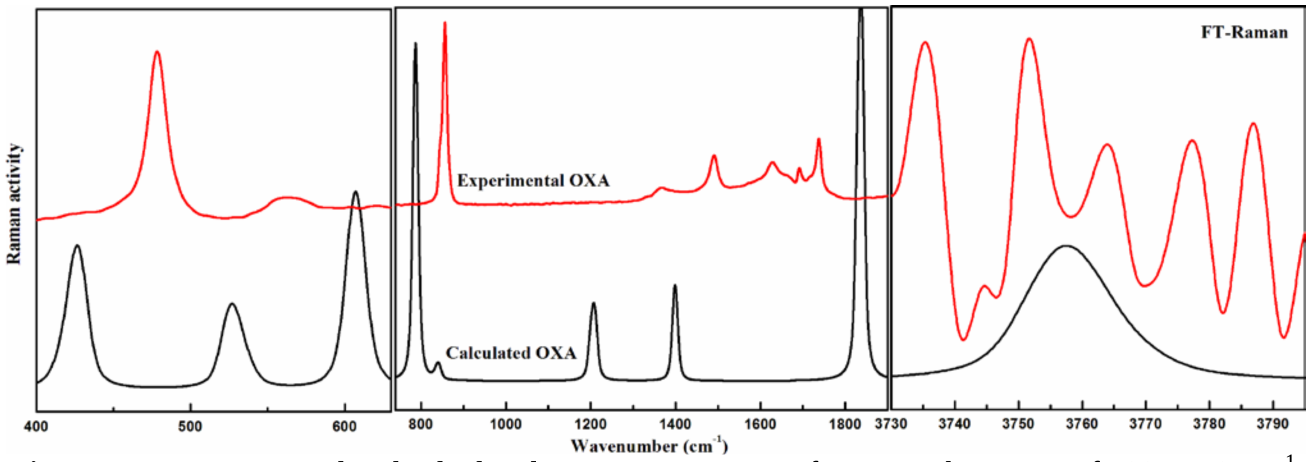

Figure S11. Experimental and calculated FT-Raman spectra of OXA in the region of 100-3560 cm^{-1}.

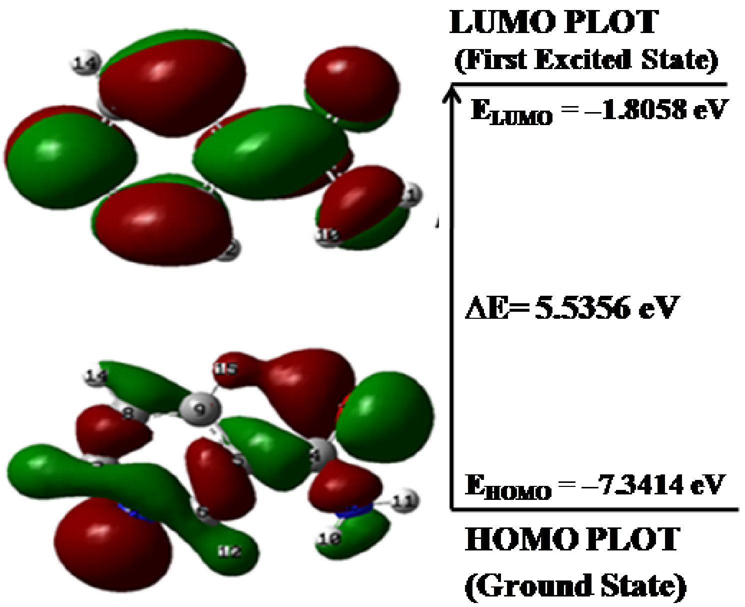

Figure S12. HOMO–LUMO energy gap of NIC with orbitals participating in electronic transitions.

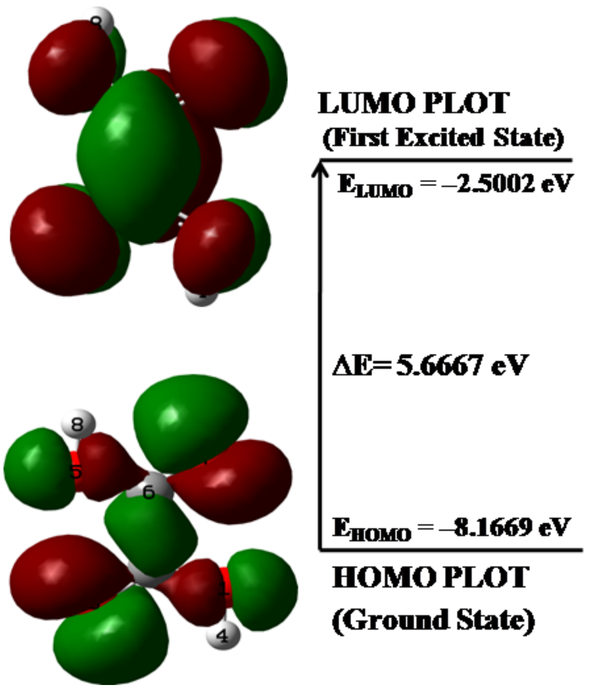

Figure S13. HOMO–LUMO energy gap of OXA with orbitals participating in electronic transitions.

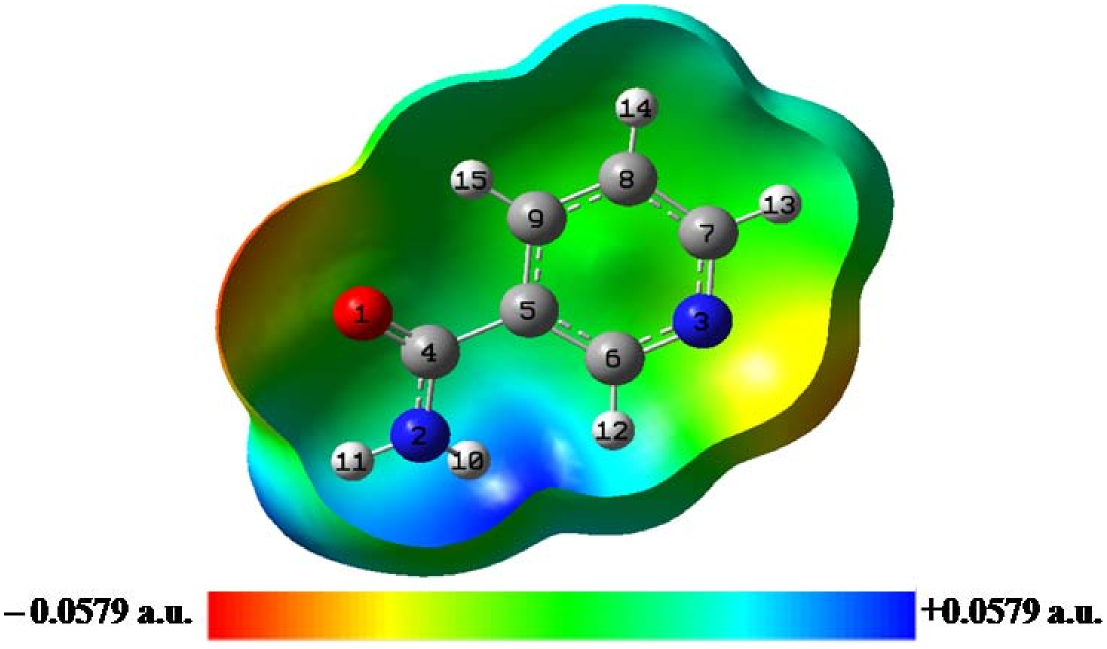

Figure S14. Molecular electrostatic potential (MESP) map of NIC formed by mapping of total electron density over electrostatic potential in gas phase.

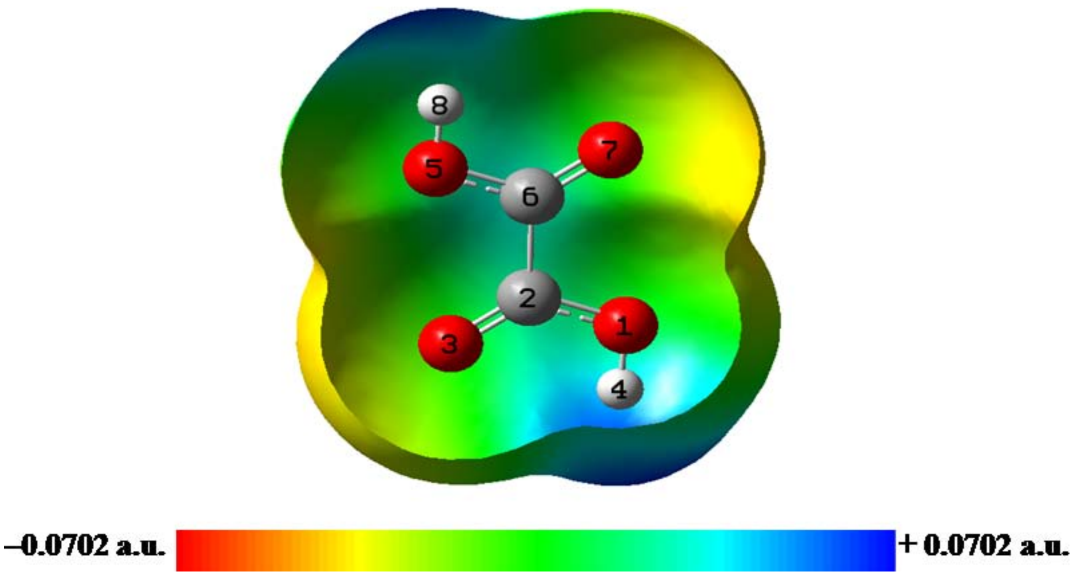

Figure S15. Molecular electrostatic potential (MESP) map of OXA formed by mapping of total electron density over electrostatic potential in gas phase.

1.2 Supplementary Tables

Table S1 The experimental and calculated geometric parameters of NIC-OXA salt and calculated geometric parameters of NIC and NIC-OXA salt using DFT/6-311++G(d,p), bond-lengths in angstroms (A) and bond-angles and dihedral-angles in degrees (˚).

Geometrical parameters	Experimental NIC	Optimized NIC	Experimental OXA	Optimized OXA	Experimental NIC-OXA salt	Optimized NIC-OXA salt
			**Bond-length (Å)**			
R(O1=C3)	-	-	1.2100	1.1988	1.2150	1.2082
R(O2-C3)	-	-	1.3071	1.3389	1.2848	1.3148
R(O2-H24)	-	-	-	-	1.7603	1.0077
R(C3-C6)	-	-	1.5378	1.5443	1.5423	1.5572
R(O4=C6)	-	-	1.2100	1.1988	1.215	1.2082
R(O5-C6)		-	1.3071	1.3389	1.2848	1.3148
R(O5-H8)	-	-	-	-	1.7603	1.0077
R(N7-H8)	-	-	-	-	0.8604	1.7536
R(N7-C10)	1.34062	1.33603	-	-	1.3371	1.3354
R(N7-C18)	1.34084	1.33502	-	-	1.3446	1.3397
R(O9=C11)	1.23728	1.21995	-	-	1.2345	1.217
R(C10-C12)	1.39569	1.399	-	-	1.3786	1.3995
R(C10-H22)	1.08304	1.0868	-	-	1.019	1.0832
R(C11-C12)	1.49796	1.5031	-	-	1.4994	1.5033
R(C11-N13)	1.34022	1.369	-	-	1.3236	1.3734

R(C12-C19)	1.39115	1.3972	-	-	1.398	1.396
R(N13-H14)	1.01029	1.0063		-	0.8602	1.0095
R(N13-H15)	1.01092	1.0087	-	-	0.8601	1.0072
R(C16-C18)	1.39122	1.395	-	-	1.373	1.3903
R(C16-C19)	1.38860	1.3879	-	-	1.3865	1.3915
R(C16-H21)	1.08259	1.0835	-	-	1.081	1.083
R(H17-C18)	1.08334	1.0862	-	-	0.997	1.0854
R(C19-H20)	1.08342	1.0833	-	-	0.9846	1.0844
R(N23-H24)	-	-	-	-	0.8604	1.7536
R(N23-C26)	1.34062	1.335	-	-	1.3371	1.3354
R(N23-C34)	1.34084	1.33502	-	-	1.3446	1.3397
R(O25=C27)	1.23728	1.2199	-	-	1.2345	1.217
R(C26-C28)	1.39569	1.399	-	-	1.3786	1.3995
R(C26-H38)	1.08304	1.0868	-	-	1.019	1.0832
R(C27-C28)	1.49796	1.5031	-	-	1.4994	1.5033
R(C27-N29)	1.34022	1.369	-	-	1.3236	1.3734
R(C28-C35)	1.39115	1.3972	-	-	1.398	1.396
R(N29-H30)	1.01029	1.0063	-	-	0.8602	1.0095
R(N29-H31)	1.01092	1.0087	-	-	0.8601	1.0072
R(C32-C34)	1.39122	1.395	-	-	1.373	1.3903
R(C32-C35)	1.38860	1.3879	-	-	1.3865	1.3915
R(C32-H37)	1.08259	1.0835	-	-	1.081	1.083
R(H33-C34)	1.08334	1.0862	-	-	0.997	1.0854
R(C35-H36)	1.08342	1.0833	-	-	0.9846	1.0844
			**Bond-angle (°)**			
A(C3-O2-H24)	-	-	-	-	124.2382	115.7501
A(O1=C3-O2)	-	-	126.9729	125.4462	125.0235	123.6763
A(O1=C3-C6)	-	-	122.4950	124.3263	119.6004	120.8511
A(O2-C3-C6)	-	-	110.5319	110.2275	115.3707	115.4725
A(C6-O5-H8)	-	-	-	-	124.2382	115.7501
A(C3-C6=O4)	-	-	122.4950	124.3263	119.6004	120.8511
A(C3-C6-O5)	-	-	110.5319	110.2275	115.3707	115.4725
A(O4=C6-O5)	-	-	126.9729	125.4462	125.0235	123.6763
A(H8-N7-C10)	-	-	-	-	119.5555	124.6425
A(H8-N7-C18)	-	-	-	-	119.5718	116.6316
A(C10-N7-C18)	117.86146	117.42614	-	-	120.8727	118.7229
A(O5-H8-N7)	-	-	-	-	158.0492	155.3597
A(N7-C10-C12)	-	-	-	-	121.1567	122.6437
A(N7-C10-H22)	-	-	-	-	117.3866	116.5627
A(C12-C10-H22)	121.35776	120.84398	-	-	121.4565	120.7919
A(O9=C11-C12)	119.26758	121.46334	-		119.0813	121.7797
A(O9=C11-N13)	123.15125	122.04163	-	-	123.6769	122.4036
A(C12-C11-N13)	117.57986	116.48896	-	-	117.2394	115.7956
A(C10-C12-C11)	124.07542	123.94345	-	-	117.7716	118.2629
A(C10-C12-C19)	118.13318	117.74034	-	-	118.4759	118.1835
A(C11-C12-C19)	117.77236	118.29810	-	-	123.7525	123.524
A(C11-N13-H14)	120.44431	121.91331	-	-	119.9904	116.0891
A(C11-N13-H15)	117.84108	116.70270	-	-	120.0341	120.9392
A(H14-N13-H15)	120.37884	117.78917	-	-	119.9755	117.0709
A(C18-C16-C19)	118.02506	118.55928	-	-	119.005	118.4548
A(C18-C16-H21)	120.28383	120.27579	-	-	122.3662	120.2874

A(C19-C16-H21)	121.66944	121.16488	-	-	118.5211	121.2556
A(N7-C18-C16)	123.41160	123.44545	-	-	120.9919	122.8272
A(N7-C18-H17)	116.22340	116.04571	-	-	122.1358	116.0709
A(C16-C18-H17)	120.36374	120.50801	-	-	116.7803	121.1015
A(C12-C19-C16)	119.50843	118.92348	-	-	119.4906	119.1608
A(C12-C19-H20)	118.69720	119.00237	-	-	121.2878	120.6859
A(C16-C19-H20)	121.78713	122.07391	-	-	119.217	120.1267
A(H24-N23-C26)	-	-	-	-	119.5555	124.6425
A(H24-N23-C34)	-	-	-	-	119.5718	116.6316
A(C26-N23-C34)	117.86146	117.42614	-	-	120.8727	118.7229
A(O2-H24-N23)	-	-	-	-	158.0492	155.3597
A(N23-C26-C28)	123.01499	123.88879	-	-	121.1567	122.6437
A(N23-C26-H38)	115.56278	115.24512	-	-	117.3866	116.5627
A(C28-C26-H38)	121.35776	120.84398	-	-	121.4565	120.7919
A(O25=C27-C28)	119.26758	121.46334	-	-	119.0813	121.7797
A(O25=C27-N29	123.15125	122.04163	-	-	123.6769	122.4036
A(C28-C27-N29)	117.57986	116.48896	-	-	117.2394	115.7956
A(C26-C28-C27)	124.07542	123.94345	-	-	117.7716	118.2629
A(C26-C28-C35)	118.13318	117.74034	-	-	118.4759	118.1835
A(C27-C28-C35)	117.77236	118.29810	-	-	123.7525	123.524
A(C27-N29-H30	120.44431	121.91331	-	-	119.9904	116.0891
A(C27-N29-H31)	117.84108	116.70270	-	-	120.0341	120.9392
A(H30-N29-H31)	120.37884	117.78917	-	-	119.9755	117.0709
A(C34-C32-C35)	121.66944	118.55928	-	-	119.005	118.4548
A(C34-C32-H37)	120.28383	120.27579	-	-	122.3662	120.2874
A(C35-C32-H37)	121.66944	121.16488	-	-	118.5211	121.2556
A(N23-C34-C32)	123.41160	123.44545	-	-	120.9919	122.8272
A(N23-C34-H33)	116.22340	116.04571	-	-	122.1358	116.0709
A(C32-C34-H33)	120.36374	120.50801	-	-	116.7803	121.1015
A(C28-C35-C32)	119.50843	118.92348	-	-	119.4906	119.1608
A(C28-C35-H36)	118.69720	119.00237	-	-	121.2878	120.6859
A(C32-C35-H36)	121.78713	122.07391	-	-	119.217	120.1267
**Dihedral-angle (˚)**						
D(H24-O2-C3=O1)	-	-	-	-	-179.741	-179.85
D(H24-O2-C3-C6)	-	-	-	-	1.1049	0.1971
D(C3-O2-H24-N23)	-	-	-	-	160.0764	177.2078
D(O1=C3-C6-O4)	-	-	-180.00000	180.00000	180	180
D(O1=C3-C6-O5	-	-	0.17400	0.00585	0.7971	0.0453
D(O2-C3-C6=O4)	-	-	-0.17400	-0.00585	-0.7971	-0.0453
D(O2-C3-C6-O5)	-	-	180.00000	180.00000	180	-180
D(H8-O5-C6-C3)	-	-	-	-	-1.1049	-0.1971
D(H8-O5-C6=O4)	-	-	-	-	179.7414	179.8497
D(C6-O5-H8-N7)	-	-	-	-	-160.076	-177.208
D(C10-N7-H8-O5)	-	-	-	-	137.2512	-174.024
D(C18-N7-H8-O5)	-	-	-	-	-42.7603	6.6111
D(H8-N7-C10-C12)	-	-	-	-	-179.115	-178.397
D(H8-N7-C10-H22	-	-	-	-	0.6927	1.1323
D(C18-N7-C10-C12)	-	-	-	-	0.8967	0.9554
D(C18-N7-C10-H22	-	-	-	-	-179.296	-179.515
D(H8-N7-C18-C16)	-	-	-	-	179.6393	179.0342
D(H8-N7-C18-H17)	-	-	-	-	3.2411	-1.1767
D(C10-N7-C18-C16)	-	-	-	-	-0.3724	-0.3702

D(C10-N7-C18-H17)	-	-	-	-	-176.771	179.419
D(N7-C10-C12-C11)	-	-	-	-	179.4129	-178.965
D(N7-C10-C12-C19)	-	-	-	-	-0.6127	-0.8733
D(H22-C10-C12-C11)	-2.19506	-1.41632	-	-	-0.3868	1.5248
D(H22-C10-C12-C19)	176.17920	177.00649	-	-	179.5876	179.6165
D(O9=C11-C12-C10)	-20.73370	159.48037	-	-	26.4713	28.7311
D(O9=C11-C12-C19)	-20.73370	-18.93425	-	-	-153.502	-149.251
D(N13-C11-C12-C10)	-22.75828	-19.64091	-	-	-152.986	-152.901
D(N13-C11-C12-C19)	158.86203	161.94448	-	-	27.041	29.1168
D(O9=C11-N13-H14)	168.59367	163.87375	-	-	-0.0025	-7.6409
D(O9=C11-N13-H15)	1.73824	5.73898	-	-	179.9774	-159.805
D(C12-C11-N13-H14)	-10.98510	-17.01048	-	-	179.4277	174.0024
D(C12-C11-N13-H15)	-177.84053	-175.14526	-	-	-0.5924	21.8378
D(C10-C12-C19-C16)	2.20657	1.33253	-	-	-0.1734	0.1973
D(C10-C12-C19-H20)	-176.83210	-178.49534	-	-	-179.387	-177.928
D(C11-C12-C19-C16)	-179.31531	179.84654	-	-	179.7993	178.181
D(C11-C12-C19-H20)	1.64601	0.01867	-	-	0.5857	0.0558
D(C19-C16-C18-N7)	-0.03287	-0.62223	-	-	-0.4134	-0.2712
D(C19-C16-C18-H17)	-179.60831	179.72244	-	-	176.1704	179.95
D(H21-C16-C18-N7)	178.30228	179.29719	-	-	-176.582	179.1934
D(H21-C16-C18-H17)	179.32693	-0.35814	-	-	0.0013	-0.5854
D(C18-C16-C19-C12)	-1.83070	-0.49541	-	-	0.6712	0.3394
D(C18-C16-C19-H20)	177.17726	179.32693	-		179.9013	178.4754
D(H21-C16-C19-C12)	179.85851	179.58591	-	-	176.9888	-179.12
D(H21-C16-C19-H20)	-1.13354	-0.59175	-	-	-3.7812	-0.9838
D(C26-N23-H24-O2)	-	-	-	-	-137.251	174.0239
D(C34-N23-H24-O2)		-	-	-	42.7603	-6.6111
D(H24-N23-C26-C28)	-	-	-	-	179.1149	178.3974
D(H24-N23-C26-H38)	-	-	-	-	-0.6927	-1.1323
D(C34-N23-C26-C28)	-1.04660	0.13315	-	-	-0.8967	-0.9554
D(C34-N23-C26-H38)	-178.15912	-178.16475	-	-	179.2957	179.515
D(H24-N23-C34-C32)	-	-	-	-	-179.639	-179.034
D(H24-N23-C34-H33)	-	-	-	-	-3.2411	1.1767
D(C26-N23-C34-C32)	1.46387	0.80539	-	-	0.3724	0.3702
D(C26-N23-C34-H33)	-178.94449	-179.52513	-	-	176.7705	-179.419
D(N23-C26-C28-C27)	-179.14452	-179.62314	-	-	-179.413	178.9649
D(N23-C26-C28-C35)	-0.77026	-1.20033	-	-	0.6127	0.8733
D(H38-C26-C28-C27)	-2.19506	-1.41632	-	-	0.3868	-1.5248
D(H38-C26-C28-C35)	176.17920	177.00649	-	-	-179.588	-179.617
D(O25=C27-C28-C26	-20.73370	159.48037	-	-	-26.4713	-28.7311
D(O25=C27-C28-C35)	-20.73370	-18.93425	-	-	153.5016	149.2512
D(N29-C27-C28-C26)	-22.75828	-19.64091	-	-	152.9861	152.901
D(N29-C27-C28-C35)	158.86203	161.94448	-	-	-27.041	-29.1168
D(O25=C27-N29-H30	168.59367	163.87375	-	-	0.0025	7.6409
D(O25=C27-N29-H31)	1.73824	5.73898	-	-	-179.977	159.8054
D(C28-C27-N29-H30)	-10.98510	-17.01048	-	-	-179.428	-174.002
D(C28-C27-N29-H31)	-177.84053	-175.14526	-	-	0.5924	-21.8378
D(C26-C28-C35-C32)	2.20657	1.33253	-	-	0.1734	-0.1973
D(C26-C28-C35-H36)	-176.83210	-178.49534	-	-	179.387	177.928
D(C27-C28-C35-C32)	-179.31531	179.84654	-	-	-179.799	-178.181
D(C27-C28-C35-H36)	1.64601	0.01867	-	-	-0.5857	-0.0558

D(C35-C32-C34-N23)	-0.03287	-0.62223	-	-	0.4134	0.2712
D(C35-C32-C34-H33)	-179.60831	179.72244	-	-	-176.17	-179.95
D(H37-C32-C34-N23)	178.30228	179.29719	-	-	176.5824	-179.193
D(H37-C32-C34-H33)	-1.27316	-1.27316	-	-	-0.0013	0.5854
D(C34-C32-C35-C28)	-1.83070	-0.49541	-	-	-0.6712	-0.3394
D(C34-C32-C35-H36)	177.17726	179.32693	-	-	-179.901	-178.475
D(H37-C32-C35-C28)	179.85851	179.58591	-	-	-176.989	179.1197
D(H37-C32-C35-H36)	-1.13354	-0.59175	-	-	3.7812	0.9838

Table S2 Theoretical and experimental vibrational wavenumbers (cm^{-1}) of NIC and their assignments using B3LYP/6-311++G(d,p).

Unscaled	Scaled	IR	Raman	Potential Energy Distribution (≥ 5%)
3715	3523			[ν(N2H10)](58)+[ν(N2H11)](41)
3589	3410	3360	3372	[ν(N2H11)](58)+[ν(N2H10)](41)
3202	3063	3060	3062	R1[ν(CH)](99)
3186	3049	3047	3034	R1[ν(CH)](99)
3155	3020	3019	3018	R1[ν(C7H13)](97)
3144	3010	3013	3009	R1[ν(C6H12)](97)
1746	1712	1674	1677	[ν(C4=O1)](71)+[ν(C4N2)](8)+[ρ(C4N2)](6)+[δ_{sci}(C4N2)](3)
1629	1600	1614	1615	R1[ν(CC)](45)+R1[ν(C6N3)](15)+R1[δ_{in}(CH)](17)+R1[δ'_{a}](8)
1621	1592	1592	1597	[δ_{sci}(C4N2)](85)+[ν(C4=O1)](5)+[ν(C4N2)](5)
1606	1578	1575	1579	R1[ν(CC)](51)+R1[ν(C7N3)](15)+R1[δ_{in}(C7H13)](12)+R1[δ_{a}](7)
1508	1484	1485	1491	R1[δ_{in}(CH)](54)+R1[ν(C7N3)](10)+R1[ν(CC)](17)
1448	1427	1421	1431	R1[δ_{in}(C7H13)](46)+R1[ν(C6N3)(14)+R1[ν(CC)](26)
1364	1346	1340	1346	[ν(C4N2)](31)+R1[ν(C4C5)](21)+[δ_{sym}(C4N2)](12)+[ρ(C4N2)](7)+ R1[δ_{in}(CH)](11)
1360	1341			R1[δ_{in}(CH)](78)
1288	1272	1264	1276	R1[ν(C6N3)](24)+R1[ν(C7N3)](19)+R1[ν(CC)(48)
1227	1213	1202	1211	R1[δ_{in}(CH)](49)+R1[ν(C7N3)](20)+R1[ν(C7C8)](11)+R1[ν(C6N3)](8)
1155	1143	1153	1139	R1[δ_{in}(C8H14)](22)+R1[ν(CC)](36)+R1[δ_{tri}](14)+[ν(C4N2)](9)+[ρ(C4N2)](9)
1132	1121	1124	1124	R1[δ_{in}(CH)](45)+R1[ν(C6N3)](14)+R1[ν(CC)](22)
1083	1074	1067	1070	[ρ(C4N2)](56)+[ν(C4N2)](24)+[ν(C4=O1)](8)
1058	1049	1038	1042	R1[ν(CC)](55)+R1[ν(C7N3)](17)+R1[δ_{in}(C8H14)](7)+R1[ν(C6N3)](6)
1039	1031	1028	1034	R1[δ_{tri}](70)+R1[ν(CC)](17)
1014	1006	995	1005	R1[oop(CH)](86)+R1[puck](8)
988	980	979	972	R1[oop(CH)](90)
947	940	935	939	R1[oop(CH)](82)+R1[puck](12)
843	839	828	834	R1[oop(CH)](51)+R1[puck](20)+[ω(C4N2)](12)+R1[oop(C4C5)](12)
780	777	775	777	R1[δ'_{a}](34)+R1[ν(CC)](30)+R1[δ_{tri}](7)+[ρ(C4N2)](5)+[ν(C4N2)](5)
750	748	732	741	[ω(C4N2)](51)+R1[oop(CH)](38)
719	717	701	705	R1[puck](79)+R1[oop(C8H14)](13)+R1[oop(C4C5)](5)

646	644	644	646	R1[δ’$_a$](32)+R1[δ$_a$](27)+[δ$_{sym}$(C4N2)](18)+[ρ(C4N2)](12)
620	619	620	629	R1[δ$_a$](48)+[ρ(C4N2)](23)+[δ$_{sym}$(C4N2)](12)
556	556	550	556	[τ(C4N2)](71)+R1[oop(C4C5)](8)+R1[τ’$_a$](5)
497	497	509	499	[ρ(C4N2)](24)+[δ$_{sym}$(C4N2)](17)+R1[δ$_{in}$(C4C5)](12)+R1[oop(C4C5)](8)+R1[τ$_a$](7)+[ρ(C4N2)](5)
417	418	411	415	R1[τ$_a$](71)+R1[oop(C4C5)](7)
385	386	-	389	R1[τ’$_a$](63)+R1[oop(C4C5)](17)
377	378	-	-	R1[ν(C4C5)](27)+[ρ(C4N2)](24)+R1[δ’$_a$](17)+R1[δ$_a$](7)
319	321	-	315	[ω(C4N2)](69)+[τ(C4N2)](17)+[ν(C4N2)](6)
213	214	-	198	R1[δ$_{in}$(C4C5)](58)+[δ$_{sym}$(C4N2)](13)+[ω(C4N2)](11)+[ρ(C4N2)](5)+[τ(C4N2)](5)
151	152	-	136	R1[oop(C4C5)](42)+R1[τ’$_a$](26)+[ω(C4N2)](9)+R1[oop(C9H15)](7)+R1[τ$_a$](5)
55	55	-	-	[τ(C4C5)](77)+R1[δ$_{in}$(C4C5)](6)+[ρ(C4N2)](5)

Table S3 Theoretical and experimental vibrational wavenumbers (cm^{-1}) of OXA and their assignments using B3LYP/6-311++G(d,p).

**Unscaled**	**Scaled**	**IR**	**Raman**	**Potential Energy Distribution (≥ 5%)**
3759	3562	3097		[ν(O1H4)](50)+[ν(O5H8)](50)
3759	3562			[ν(O5H8)](50)+[ν(O1H4)](50)
1836	1797	1749	1737	[ν(C2=O3)](38)+[ν(C6=O7)](38)+[ν(C2C6)](7)+[δ (O7=C6-O5)(4)+ δ (O3=C2-C6)(4)
1826	1788	1689	1691	[ν(C6=O7)](44)+[ν(C2=O3)](44)+[ν(C6O5)](4)+ [ν(C2O1)](4)
1399	1379	1367	1369	[ν(C2C6)](19)+[ν(C6O5)](17)+[ν(C2O1)](17)+ [δ$_{sym}$(C2C6)](10)+[δ(C6H8O5)](10)+[ν(C6O5)](10)+ [δ$_{sym}$(C2C6)](9)+[ρ(C2C6)](5)
1324	1307	1346	1340	[δ(C2H4O1)](31)+[δ(C6H8O5)](31)+[ν(C2O1)](10)+ [ν(C6O5)](10)+[δ$_{sym}$(C2C6)](8)+[ρ(C6O5)](6)
1206	1193	1161	1158	[δ(C6H8O5)](27)+[δ(C2H4O1)](27)+[ν(C6O5)](15) + [ν(C2O1)](15)+[ν(C2C6)](5)
1138	1127		1110	[ν(C2O1)](32)+[ν(C6O5)](32)+[δ(C6H8O5)](14)+ [δ(C2H4O1)](14)
839	835	868	856	[ω(C6O5)](49)+[ω(C2C6)](49)
787	784	783	781	[ν(C2C6)](38)+[δ$_{sym}$(C2C6)](14)+[ρ(C6O5)](12)+ [ν(C6O5)](12)+[ν(C2O1)](12)+[δ(C2H4O1)](5)+ [δ(C6H8O5)](5)
664	663	675	676	[τ(C6O5)](40)+[τ(C2O1)](40)+[ω(C2C6)](10)+ [ω(C6O5)](10)
641	640	652		[δ$_{sym}$(C2C6)](43)+[ρ(C6O5)](29)+[δ$_{sym}$(C2C6)](11)+[δ(C6H8O5)](7)+[δ(C2H4O1)](7)
607	607		620	[τ(C2O1)](49)+[τ(C6O5)](49)
529	529	517	522	[ρ(C2C6)](39)+[δ$_{sym}$(C2C6)](30)+[ρ(C6O5)](13)+[ν(C2C6)](8)+[ν(C2O1)](3)+[ν(C6O5)](3)

425	426	494	477	[ν(C2C6)](34)+[δ_{sym}(C2C6)](30)+[ρ(C6O5)](26) +[δ_{sym}(C2C6)](3)
419	419	415	397	[ω(C2C6)](41)+[ω(C6O5)](41)+[τ(C6O5)](9) +[τ(C2O1)](9)
265	266	-	281	[ρ(C2C6)](48)+[δ_{sym}(C2C6)](36)+[ρ(C6O5)](16)
5	5	-	-	[τ(C6O5)](25)+[τ(C2O1)](25)+[δ_{sym}(C2C6)](14)+ [δsym(C2C6)](10)+[ρ(C2C6)](7)+[ω(C6O5)](6)+ [ω(C2C6)](6)

Table S3 Theoretical and experimental vibrational wavenumbers (cm^{-1}) of NIC-OXA salt and their assignments using B3LYP/6-311++G(d,p).

**Unscaled**	**Scaled**	**IR**	**Raman**	**Potential Energy Distribution (≥ 5%)**
3703	3511			ν[N13H15](29)+ν[N29H31](29)+ν[N13H14](21)+ν[N29H30](21)
3703	3511			ν[N29H31](29)+ν[N13H15](29)+ν[N29H30](21)+ν[N13H14](21)
3579	3401	3379	3360	ν[N29H30](29)+ν[N13H14](29)+ν[N29H31](21)+ν[N13H15](21)
3579	3401			ν[N13H14](29)+ ν[N29H30](29)+ν[N13H15](21)+ ν[N29H31](21)
3211	3071	3095	3102	ν[C10H22](45)+ν[C26H38](38)+ν[O1H22](10)
3211	3071			ν[C10H22](45)+ν[C26H38](38)+ν[O1H22](10)
3198	3059			ν[C32H37](41)+ν[C16H21](41)+ν[C19H20](5)+ν[C35H36](5)
3198	3059			ν[C32H37](41)+ν[C16H21](41)+ν[C35H36](5)+ ν[C19H20](5)
3175	3038			ν[C19H20](39)+ν[C35H36](39)+ν[C18H17](8)+ν[C34H33](8)
3175	3038			ν[C35H36](39)+ν[C19H20](39)+ν[C34H33](8)+ ν[C18H17](8)
3168	3032			ν[C18H17](36)+ν[C34H33](36)+ν[C16H21](6)+ν[C32H37](6)+ν[C19H20](5)+ν[C35H36](5)
3168	3032			ν[C34H33](36)+ν[C18H17](36)+ν[C32H37](6)+ν[C16H21]6)+ν[C35H36](5)+ν[C19H20](5)
3020	2897	2852	-	ν[N23H24](33) +ν[O2H24](26)+ν[O5H8](20)+ R1[δ_{in}(N7H8)](9)+R2[δ_{in}(N23H24)](9)
3016	2894			ν[N7H8](32)+ν[O2H24](25)+ν[O5H8](20)+R1[δ_{in}(N7H8)](10)+R2[δ_{in}(N23H24)](10)
1809	1772			δ[C3H24O2](22)+ν[C3=O1](17)+ν[C6=O4](14)+δ[C6H8O5](11)+ν[C6O5](11)+ν[C3O2](8)
1796	1759	1695	1689	δ[C3H24O2](18) +ν[C3=O1](13)+[ρ(C6O5)](13)+ ν[C6=O4](11)+δ[C6H8O5](9)+ν[C6O5](6)+[δ_{in}(C3O2](6) +δ[C6O5N7](5)+ν[C3O2](5)
1758	1723			ν[C11=O9](36)+ν[C27=O25](36)
1757	1722	1647	1645	ν[C11=O9](36)+ν[C27=O25](36)
1633	1603	1607	1600	R1[δ_{in}(C10H22](12)+ν[C10N7](8)+R1[δ_{in}(N7H8)](6) +

				R2[δ_{in}(N23H24)](6)+R2[δ_{in}(C26H38)](6)+ν[C16C19](6)+R1[δ_{a}](6)+ν[C32C35](5)+τ[N23O2](5)+ν[C26N23](5)
1632	1603			R1[δ_{in}(C10H22](12)+ν[C10N7](8)+R1[δ_{in}(N7H8)](7)+R2[δ_{in}(N23H24)](7)+R2[δ_{in}(C26H38)](6)+ν[C16C19](6)+R1[δ_{a}](5)+ν[C32C35](5)+τ[N23O2](5)+ ν[C26N23](5)
1622	1593		1594	[δ_{sym}(C11N13)](42)+[δ_{sym}(C27N29)](42)
1622	1593			[δ_{sym}(C27N29)](41)+[δ_{sym}(C11N13)](41)
1617	1588	1568		ν[C34N23](18)+τ[N23O2](14)+ν[C12C19](7)+δ[C10N7O5](6)+ν[C28C35](6)+ν[C18N7](5)
1616	1588			ν[C34N23](18)+τ[N23O2](14)+ν[C12C19](7)+ ν[C28C35](6)+δ[C10N7O5](6)+ν[C18N7](5)
1503	1479	1487	1469	R1[δ_{in}(C10H22](17)+τ[N23O2](16)+ν[C34N23](9)+R2[δ_{in}(C26H38)](8)+ν[O1H22](6)+R2[δ_{in}(C32H37)](5)+R1[δ_{in}(C16H21](5)
1503	1479			R1[δ_{in}(C10H22](17)+τ[N23O2](16)+ν[C34N23](9)+ R2[δ_{in}(C26H38)](8)+ν[O1H22](6)+R1[δ_{in}(C16H21](5)+R2[δ_{in}(C32H37)](5)
1460	1438	1458	1457	R1[δ_{in}(N7H8)](25)+R2[δ_{in}(N23H24)](24)+δ[C10N7O5](11)+τ[N23O2](10)
1458	1436			R1[δ_{in}(N7H8)](25)+R2[δ_{in}(N23H24)](24)+δ[C3H24O2](10)+δ[C10N7O5](8)+τ[N23O2](6)+δ[C6H8O5](5)
1409	1389	1404	1372	δ[C3H24O2](26)+δ[C6O5N7](16)+δ[C3O2N23](14)+ δ[C6H8O5](13)+ν[C6O5](9)+ν[C3O2](7)
1358	1340	1340	1337	R1[δ_{in}(C10H22](26)+ν[O1H22](15)+R2[δ_{in}(C26H38)](13)+ν[O4H38](5)
1358	1340			δ[C3H24O2](14)+R1[δ_{in}(C10H22](13)+ν[O1H22](8)+ δ[C6H8O5](7)+R2[δ_{in}(C26H38)](6)+R1[δ_{in}(N7H8)](5)+ R2[δ_{in}(N23H24)]5)
1350	1332		1330	R1[δ_{in}(C10H22](27)+ν[O1H22](15)+R2[δ_{in}(C26H38)](13)+ν[O4H38](6)
1348	1330	1321	1319	δ[C3H24O2](34)+δ[C6H8O5](17)+R1[δ_{in}(N7H8)](12)+ R2[δ_{in}(N23H24)](12)+δ[C6O5N7](6)+δ[C3O2N23](5)
1302	1286	1290	1284	R1[δ_{in}(C10H22](19)+ν[O1H22](12)+R2[δ_{in}(C26H38)](9)+ R2[δ_{in}(C35H36)](6)+R1[δ_{in}(C19H20](6)
1291	1275			R1[δ_{in}(N7H8)](13)+τ[N23O2](13)+R2[δ_{in}(N23H24)](13)+ ν[C34N23](9)+δ[C10N7O5](7)+δ[C3H24O2](6)+ν[C10N7](5)
1284	1268	1267	1260	δ[C3H24O2](37)+δ[C6H8O5](19)+R1[δ_{in}(N7H8)](15)+ R2[δ_{in}(N23H24)](15)+ν[N23H24](6)
1259	1244			δ[C3H24O2](26)+δ[C6H8O5](13)+δ[C6O5N7](13)+δ[C3O2N23](11)+τ[N23O2](8)
1232	1218	1220	1222	δ[C3H24O2](19)+R1[δ_{in}(N7H8)](13)+R2[δ_{in}(N23H24)](13)+δ[C6O5N7](12)+δ[C3O2N23](10)+δ[C6H8O5]

				(10)+ν[C6O5](8)+ν[N23H24](6)+ν[C3O2](6)
1222	1209			ν[O1H22](10)+δ[C3H24O2](10)+R1[δ_{in}(C10H22](10)+τ[N23O2](9)+ν[C34N23](9)+δ[C6H8O5](5)+R2[δ_{in}(C26H38)](5)
1219	1206			δ[C3H24O2](23)+δ[C6H8O5](12)+R1[δ_{in}(N7H8)](9)+R2[δ_{in}(N23H24)](9)+ν[O1H22](7)+R1[δ_{in}(C10H22](6)+ν[N23H24](5)
1155	1144			R1[δ_{tri}](11)+ν[O1H22](10)+R2[δ_{tri}](9)+R1[δ_{in}(C10H22](7)+R1[δ_{in}(C16H21](6)+R2[δ_{in}(C32H37)](6)+ν[C16C19](5)+ν[C32C35](5)
1155	1143	1143	1139	R1[δ_{tri}](11)+ν[O1H22](9)+R2[δ_{tri}](9)+R1[δ_{in}(C10H22](7)+R2[δ_{in}(C32H37)](6)+R1[δ_{in}(C16H21](6)+ν[C16C19](6)+ν[C32C35](5)
1140	1129			δ[C3H24O2](20)+ν[N23H24](11)+δ[C6H8O5](10)+δ[C10N7O5](7)+R2[δ_{in}(C35H36)](6)+R1[δ_{in}(C19H20](6)+ν[C10N7](6)
1139	1128	1128	1118	δ[C3H24O2](11)+ν[N23H24](11)+δ[C10N7O5](8)+R1[δ_{in}(C19H20](8)+R2[δ_{in}(C35H36)](8)+ν[C10N7](7)+δ[C6H8O5](6)+ν[C34N23](5)
1086	1076	1082	1077	[ρ(C11N13)](22)+[ρ(C27N29)](22)+ν[C11N13](12)+ν[C27N9](12)
1086	1076			[ρ(C27N29)](21)+[ρ(C11N13)](21)+ν[C27N9](11)+ν[C11N13](11)+δ[C3H24O2](5)
1065	1056			τ[N23O2](35)+ν[C34N23](12)+R1[δ_{in}(N7H8)](7)+R2[δ_{in}(N23H24)]7)
1064	1055			R1[δ_{tri}](24)+δ[C10N7O5](20)+R2[δ_{tri}](19)+ν[O1H22](8)+τ[N23O2](5)
1045	1036	1035	1043	τ[N23O2](36)+ν[C34N23](12)+R1[δ_{in}(N7H8)](8)+R2[δ_{in}(N23H24)]8)
1044	1036			R1[δ_{tri}](25)+δ[C10N7O5](20)+R2[δ_{tri}](20)+ν[O1H22](8)
1021	1013	1020		τ[C6O5](19)+R1[oop(C10H22](15)+R1[δ_{in}(N7H8)](14)+R2[δ_{in}(N23H24)](14)+R2[oop(C26H38)](12)+R1[oop(N7H8)](5)
1019	1011			R1[oop(C10H22](17)+τ[C6O5](17)+R2[oop(C26H38)](14)+R1[δ_{in}(N7H8)](13)+R2[δ_{in}(N23H24)](13)+ν[O1H22](5)
1010	1002	1002		R1[oop(C10H22](11)+R1[oop(C18H17](10)+R2[oop(C34H33)](10)+R2[oop(C26H38)](9)+τ[C6O5](8)+R1[oop(C16H21](8)+R2[oop(C32H37)](8) R1[δ_{in}(N7H8)](6)+ R2[δ_{in}(N23H24)](6)+ν[O1H22](5)
1010	1002			τ[C6O5](11)+R2[oop(C34H33)](10)+R1[oop(C18H17](10)+R1[oop(C10H22](8)+R1[δ_{in}(N7H8)](8)+R2[δ_{in}(N23H24)]8)+R2[oop(C32H37)](7)+R1[oop(C16H21](7)+R2[oop(C26H38)](7)
987	979	975	979	R2[oop(C35H36)](16)+R1[δ_{in}(C19H20](16)+R1[oop(C10H22](14)+R2[oop(C26H38)](12)+R2[oop(C32H37)](9)+

				R1[oop(C16H21](9)+R1[puck](6)+R2[puck](5)
986	979			R1[δ_{in}(C19H20](16)+R2[oop(C35H36)](16)+R1[oop(C10H22](14)+R2[oop(C26H38)](12)+R1[oop(C16H21](9)+R2[oop(C32H37)](9)+R1[puck](6)+R2[puck](5)
967	960	948	952	R1[oop(C18H17](22)+R2[oop(C34H33)](22)+R1[oop(C10H22](10)+R2[oop(C26H38)](8)+R1[δ_{in}(C19H20](8)+R2[oop(C35H36)](8)
967	960			R2[oop(C34H33)](22)+R1[oop(C18H17](22)+R1[oop(C10H22](10)+R2[oop(C26H38)](9)+R2[oop(C35H36)](8)+ R1[δ_{in}(C19H20](8)
851	847	844	853	[ρ(C6O5)](27)+[δ_{in}(C3O2](12)+ν[C6O5](9)+R1[δ_{in}(N7H8)](8)+R2[δ_{in}(N23H24)](8)+ν[C3O2](6)+ν[C3C6](6)
835	831		833	R1[puck](11)+R1[oop(C16H21](10)+R2[oop(C32H37)](10)+R2[puck](10)+R1[oop(C18H17](8)+R2[oop(C34H33)](8)+R1[δ_{in}(C19H20](7)+R2[oop(C35H36)](7)+[ω(C11N13)](6)+[ω(C27N29)](6)+R1[oop(C11C12)](6)+R2[oop(C27C28)](6)
835	831			R1[puck](11)+R2[oop(C32H37)](10)+R1[oop(C16H21](10)+R2[puck](10)+R2[oop(C34H33)](8)+R1[oop(C18H17](8)+R2[oop(C35H36)](7)+R1[δ_{in}(C19H20](7)+[ω(C27N29)](6)+[ω(C11N13)](6)+R2[oop(C27C28)](6)+R1[oop(C11C12)](6)
810	806			[ω(C6O5)](43)+[oop(C3O2](41)+τ[C6O5](6)+τ[N7O5](5)
786	782	786	786	ν[N23H24](18)+R1[δ_{in}(N7H8)](8)+R2[δ_{in}(N23H24)](8)+ R1[δ'_{a}](7)+R2[δ'_{a}](6)+R1[δ_{a}](6)
784	781			ν[N23H24](14)+R1[δ'_{a}](7)+R2[δ'_{a}](6)+δ[C6O5N7](6)+τ[N23O2](6)+R1[δ_{a}](5)+δ[C3O2N23](5)
755	753	738	741	[ω(C11N13)](22)+[ω(C27N29)](22)+R1[oop(C16H21](6)+ R2[oop(C32H37)](6)+R1[δ_{in}(C19H20](5)+R2[oop(C35H36)](5)
755	752			[ω(C27N29)](23)+[ω(C11N13)](23)+R2[oop(C32H37)](6)+R1[oop(C16H21](6)+R2[oop(C35H36)](6)+R1[δ_{in}(C19H20](6)
721	718			[ρ(C6O5)](24)+δ[C6O5N7](20)+δ[C3O2N23](17)+[δ_{in}(C3O2](10)+R1[δ_{in}(N7H8)](8)+R2[δ_{in}(N23H24)]8)
715	713	711	711	R1[puck](36)+R2[puck](35)+R1[oop(C16H21](6)+R2[oop(C32H37)](6)
715	713			R1[puck](36)+R2[puck](34)+R2[oop(C32H37)](6)+R1[oop(C16H21](6)
655	654			R1[δ_{a}](27)+ν[N23H24](19)+R2[δ_{a}](16)+δ[C10N7O5](10) +δ[C6O5N7](6)+δ[C3O2N23](5)
653	652	650	651	R1[δ_{a}](28)+ν[N23H24](18)+R2[δ_{a}](17)+δ[C10N7O5](10)
627	626	628	620	R1[δ_{a}](19)+R2[δ_{a}](11)+δ[C6O5N7](7)+[δ_{sym}(C27N29)

				](6)+[δ_{sym}(C11N13)](6)+ν[N23H24](6)+δ[C3O2N23](6)+δ[C10N7O5](5)+[ρ(C27N29)](5)+[ρ(C11N13)](5)
627	626			R1[δ_{a}](20)+R2[δ_{a}](12)+[δ_{sym}(C11N13)](6)+[δ_{sym}(C27N29)](6)+δ[C6O5N7](6)+δ[C10N7O5](5)+δ[C3O2N23](5)+ν[N23H24](5)+[ρ(C11N13)](5)+[ρ(C27N29)](5)
564	563		589	δ[C6O5N7](35)+δ[C3O2N23](30)+R1[δ_{in}(N7H8)](8)+R2[δ_{in}(N23H24)](8)
559	559			τ(C11N13](25)+[τ(C27N29)](25)
559	558	555	553	[τ(C27N29)](17)+τ(C11N13](17)+δ[C6O5N7](10)+δ[C3O2N23](8)
519	519	503	512	R1[δ_{in}(N7H8)](13)+R2[δ_{in}(N23H24)](13)+δ[C10N7O5](10)+ν[O1H22](9)
519	519			R1[δ_{in}(N7H8)](12)+R2[δ_{in}(N23H24)](12)+δ[C10N7O5](10)+ν[O1H22](8)+ν[N23H24](5)
468	468	476	472	[ω(C6O5)](40)+[oop(C3O2](39)+τ[N7O5](12)
438	438	449	443	δ[C6O5N7](26)+δ[C3O2N23](22)+R1[δ_{in}(N7H8)](17)+ R2[δ_{in}(N23H24)](17)+[ρ(C6O5)](5)
418	419	420	412	R1[τ_{a}](28)+R2[τ_{a}](26)+τ[N7O5](8)+R1[oop(N7H8)](5)
418	419			R1[τ_{a}](25)+R2[τ_{a}](23)+τ[N7O5](9)+R1[oop(N7H8)](5)
394	395	-		[ω(C27N29)](20)+[ω(C11N13)](20)+R1[τ'_{a}](13)+R2[τ'_{a}](12)+R2[oop(C27C28)](5)+ R1[oop(C11C12)](5)
393	394	-		[ω(C11N13)](20)+[ω(C27N29)](20)+R1[τ'_{a}](13)+R2[τ'_{a}](12)+R1[oop(C11C12)](5)+R2[oop(C27C28)](5)
379	380	-		R1[τ'_{a}](13)+R2[τ'_{a}](12)+[ω(C11N13)](10)+[ω(C27N29)](10)+τ(C11N13](5)+[τ(C27N29)](5)+R1[δ_{in}(N7H8)](5)+R2[δ_{in}(N23H24)](5)
379	380	-		R1[τ'_{a}](14)+R2[τ'_{a}](14)+[ω(C27N29)](12)+[ω(C11N13)](12)+[τ(C27N29)](6)+τ(C11N13](6)
370	371	-		δ[C10N7O5](16)+ν[N23H24](8)+R1[δ_{a}](7)+R1[δ_{in}(N7H8)](7)+R2[δ_{in}(N23H24)](6)
368	369	-	364	δ[C6O5N7](17)+δ[C3O2N23](14)+R1[δ_{in}(N7H8)](13)+R2[δ_{in}(N23H24)](13)+δ[C10N7O5](11)
309	310	-	306	δ[C6O5N7](34)+δ[C3O2N23](29)+R1[δ_{in}(N7H8)](13)+R2[δ_{in}(N23H24)](13)
221	223	-	240	R1[δ_{in}(N7H8)](26)+R2[δ_{in}(N23H24)](25)+δ[C10N7O5](17)+δ[C6O5N7](5)+ν[N23H24](5)
215	216	-	202	δ[C10N7O5](19)+R1[δ_{in}(N7H8)](18)+R2[δ_{in}(N23H24)](18)+ν[O1H22](12)+τ[N23O2](11)
164	165	-	180	δ[C6O5N7](25)+δ[C3O2N23](21)+ν[N23H24](15)+ν[O1H22](7)+δ[C3H24O2](6)+τ[N23O2](6)+R1[δ_{in}(N7H8)](5)+ R2[δ_{in}(N23H24)](5)
152	153	-		R1[δ_{in}(N7H8)](15)+R2[δ_{in}(N23H24)](15)+τ[N23O2](15)+τ[N7O5](12)+δ[C10N7O5](9)+R1[oop(C11C12)](5)+R2[oop(C27C28)](5)
151	152	-	144	τ[N23O2](23)+ν[N23H24](15)+δ[C6O5N7](10)+τ[N7

				O5](9)+δ[C3O2N23](8)+R2[oop(C27C28)](5)+R1[(oop(C11C12)](5)
124	125	-	124	ν[N23H24](23)+δ[C6O5N7](20)+τ[N23O2](17)+δ[C3O2N23](17)+R1[δ_{in}(N7H8)](8)+R2[δ_{in}(N23H24)](8)
115	116	-	119	τ[N7O5](39)+τ[C6O5](18)+δ[C6O5N7](13)+δ[C3O2N23](11)
103	103	-		R1[δ_{in}(N7H8)](27)+R2[δ_{in}(N23H24)](27)+δ[C10N7O5](17)+τ[N23O2](8)+δ[C3H24O2](5)
93	94	-	-	τ[N7O5](28)+R1[δ_{in}(N7H8)](14)+R2[δ_{in}(N23H24)](14)+τ[C6O5](10)+ν[O1H22](7)+δ[C10N7O5](7)+τ[N23O2](6)+R1[oop(N7H8)](5)
78	79	-	-	τ[N23O2](25)+R1[δ_{in}(N7H8)](17)+R2[δ_{in}(N23H24)](17)+ν[N23H24](13)+δ[C6O5N7](13)+δ[C3O2N23](11)
74	74	-	-	ν[O1H22](21)+R1[δ_{in}(N7H8)](16)+R2[δ_{in}(N23H24)](16)+τ[N23O2](12)+δ[C10N7O5](9)+ν[O4H38](8)
59	60	-	-	τ[N7O5](18)+δ[C6O5N7](16)+δ[C3O2N23](14)+τ[N23O2](11)+τ[N23O2](7)+τ(C11C12](6)+[τ(C27C28)](6)+τ[C6O5](5)
41	41	-	-	ν[O1H22](23)+τ[N23O2](15)+R1[δ_{in}(N7H8)](10)+R2[δ_{in}(N23H24)](10)+ν[O4H38](8)+δ[C10N7O5](5)+R1[oop(N7H8)](5)
41	42	-	-	τ[N23O2](22)+δ[C10N7O5](18)+R1[δin(N7H8)](15)+R2[δ_{in}(N23H24)](15)+τ[N7O5](15)+R1[oop(N7H8)]7)
31	32	-	-	ν[O1H22](24)+R1[δ_{in}(N7H8)](15)+R2[δ_{in}(N23H24)](15)+δ[C10N7O5](13)+τ[N23O2](13)+ν[O4H38](9)+δ[C6O5N7](5)
23	23	-	-	τ[N7O5](20)+τ[N23O2](17)+R1[δin(N7H8)](14)+δ[C10N7O5](14)+R2[δ_{in}(N23H24)](14)+δ[C6O5N7](7)+τ[N23O2](7)+δ[C3O2N23](6)
23	23	-	-	τ[N23O2](24)+ν[O1H22](20)+R1[δ_{in}(N7H8)](13) R2[δ_{in}(N23H24)](13)+δ[C10N7O5](8)+ν[O4H38](7)+δ[C6O5N7](6)+δ[C3O2N23](5)
9	9	-	-	τ[N23O2](26)+δ[C10N7O5](18)+R1[δ_{in}(N7H8)](17)+R2[δ_{in}(N23H24)](17)+τ[N7O5](10)
6	6	-	-	τ[N23O2](20)+τ[N7O5](16)+R1[δ_{in}(N7H8)](14)+R2[δ_{in}(N23H24)](14)+δ[C10N7O5](11)+δ[C6O5N7](8)+τ[N23O2](7)+δ[C3O2N23](7)

Table S4 Geometrical parameters for intermolecular interaction in NIC-OXA salt bond distance (Å), bond-angle (°) and sum of van der Waals radii of interacting atoms (Å).

**Interactions (D−H⋯A)**	d_{D-H}	$d_{H\cdots A}$	$d_{D\cdots A}$	**D−H⋯A**	$(r_H + r_A)$
N7-H8⋯O5	1.75359	1.00767	2.70236	155.3597	2.72
C10-H22⋯O1	1.08317	2.41655	3.14145	123.0590	2.72

N23-H24···O2	1.75359	1.00767	2.70236	155.3597	2.72
C26-H38···O4	1.08317	2.41655	3.14145	123.0590	2.72

Table S5 Second-order perturbation theory analyses of the Fock Matrix, in the NBO basis for intra- and intermolecular interactions in NIC-OXA salt.

**Donor NBO(i)**	**ED(i)/e**	**Acceptor NBO(j)**	**ED(j)/e**	**$E^{(2)a}$ (kcalmol^{-1})**	**E(j)-E(i)^{b}(a.u.)**	**F(i,j)^{c} (a.u.)**
**within unit 1 (oxalic acid)**						
π(O1=C3)	1.98179	π*(O4=C6)	0.23864	4.89	0.39	0.041
σ(O2-H24)	1.98237	σ*(O1=C3)	0.02087	5.36	1.58	0.034
π(O4=C6)	1.98179	π*(O1=C3)	0.23864	4.89	0.39	0.041
σ(O5-H8)	1.98237	σ*(O4=C6)	0.02087	5.36	1.58	0.034
LP(2)O1	1.85471	σ*(O2-C3)	0.07772	27.36	19.27	0.099
LP(2)O1	1.85471	σ*(C3-C6)	0.14182	21.89	1.02	0.036
LP(1)O2	1.96616	σ*(C3-C6)	0.14182	6.16	0.58	0.101
LP(2)O2	1.76729	π*(O1=C3)	0.23864	56.78	0.33	0.121
LP(2)O4	1.85471	σ*(C3-C6)	0.14182	21.89	1.02	0.036
LP(2)O4	1.85471	σ*(O5-C6)	0.07772	27.36	1.12	0.032
LP(1)O5	1.96616	σ*(C3-C6)	0.14182	6.16	0.58	0.101
LP(2)O5	1.76729	π*(O4=C6)	0.23864	56.78	0.33	0.121
**from unit 1 (oxalic acid) to unit 2 (nicotinamide)**						
σ(O5-H8)	1.98237	σ*(N7-C10)	0.01502	0.23	1.28	0.015
σ(O5-H8)	1.98237	σ*(N7-C18)	0.01802	0.08	1.26	0.009
LP(1)O1	1.98138	σ*(C10-C12)	0.02808	0.13	1.24	0.012
LP(1)O1	1.98138	σ*(C10-H22)	0.02018	0.61	1.14	0.024
LP(2)O1	1.85471	σ*(N7-C18)	0.01802	0.08	0.78	0.007
LP(2)O1	1.85471	σ*(C10-C12)	0.02808	0.12	0.81	0.009
LP(2)O5	1.76729	π*(N7-C10)	0.34834	0.12	0.30	0.006
**from unit 1 (oxalic acid) to unit 3 (nicotinamide)**						
σ(O2-H24)	1.98237	σ*(N23-C26)	0.01502	0.23	1.28	0.015
σ(O2-H24)	1.98237	σ*(N23-C34)	0.01802	0.08	1.26	0.009
LP(2)O2	1.76729	π*(N23-C26)	0.34834	0.12	0.30	0.006
LP(1)O4	1.98138	σ*(C26-C28)	0.02808	0.13	1.24	0.012
LP(1)O4	1.98138	σ*(C26-H38)	0.02018	0.61	1.14	0.024
LP(2)O4	1.85471	σ*(N23-C34)	0.01802	0.08	0.78	0.007
LP(2)O4	1.85471	σ*(C26-C28)	0.02808	0.12	0.81	0.009
**from unit 2 (nicotinamide) to unit 1 (oxalic acid)**						
σ(N7-C10)	1.98699	σ*(O5-H8)	0.07583	0.38	1.24	0.020
σ(N7-C18)	1.98744	σ*(O5-H8)	0.07583	0.44	1.24	0.021
σ(C10-C12)	1.97698	σ*(O5-H8)	0.07583	0.16	1.13	0.012
σ(C16-C18)	1.98420	σ*(O5-H8)	0.07583	0.16	1.13	0.012
LP(1)O5	1.87249	σ*(N7-H8)	0.07583	27.39	0.76	0.131
**within unit 2 (nicotinamide)**						
π(N7-C10)	1.72165	π*(C12-C19)	0.33659	11.90	0.33	0.056
π(N7-C10)	1.72165	π*(C16-C18)	0.28606	27.11	0.33	0.084
σ(C10-H22)	1.97824	σ*(N7-C18)	0.01802	5.34	1.04	0.067
π(C12-C19)	1.64086	π*(N7-C10)	0.34834	28.70	0.27	0.079
π(C12-C19)	1.64086	π*(O9=C11)	0.25959	14.56	0.32	0.063
π(C12-C19)	1.64086	π*(C16-C18)	0.28606	16.33	0.29	0.062
π(C16-C18)	1.62500	π*(N7-C10)	0.34834	15.44	0.27	0.058

π(C16-C18)	1.62500	π*(C12-C19)	0.33659	23.76	0.28	0.074
σ(H17-C18)	1.98121	σ*(N7-C10)	0.01502	5.03	1.07	0.065
LP(1)N7	1.87249	σ*(C10-C12)	0.02808	8.12	0.91	0.079
LP(1)N7	1.87249	σ*(C16-C18)	0.02302	7.98	0.92	0.078
LP(2)O9	1.86423	σ*(C11-C12)	0.07086	19.17	0.66	0.102
LP(2)O9	1.86423	σ*(C11-N13)	0.06965	25.29	0.69	0.120
LP(1)N13	1.76918	π*(O9=C11)	0.25959	39.96	0.34	0.104
**from unit 3 (nicotinamide) to unit 1 (oxalic acid)**						
σ(N23-C26)	1.98699	σ*(O2-H24)	0.07583	0.38	1.24	0.020
σ(N23-C34)	1.98744	σ*(O2-H24)	0.07583	0.44	1.24	0.021
σ(C26-C28)	1.97698	σ*(O2-H24)	0.07583	0.16	1.12	0.012
σ(C32-C34)	1.98420	σ*(O2-H24)	0.07583	0.16	1.13	0.012
LP(1)O2	1.87249	σ*(N23-H24)	0.07583	27.39	0.76	0.131
**within unit 3 (nicotinamide)**						
π(N23-C26)	1.72165	π*(C28-C35)	0.33659	11.90	0.33	0.056
π(N23-C26)	1.72165	π*(C32-C34)	0.28606	27.11	0.33	0.084
σ(C26-H38)	1.97824	σ*(N23-C34)	0.01802	5.34	1.04	0.067
π(C28-C35)	1.64086	π*(N23-C26)	0.34834	28.70	0.27	0.079
π(C28-C35)	1.64086	π*(O25=C27)	0.25959	14.56	0.32	0.063
π(C28-C35)	1.64086	π*(C32-C34)	0.28606	16.33	0.29	0.062
π(C32-C34)	1.62500	π*(N23-C26)	0.34834	15.44	0.27	0.058
π(C32-C34)	1.62500	π*(C28-C35)	0.33659	23.76	0.28	0.074
σ(H33-C34)	1.98121	σ*(N23-C26)	0.01502	5.03	1.07	0.065
LP(1)N23	1.87249	σ*(C26-C28)	0.02808	8.12	0.91	0.079
LP(1)N23	1.87249	σ*(C32-C34)	0.02302	7.98	0.92	0.078
LP(2)O25	1.86423	σ*(C27-C28)	0.07086	19.17	0.66	0.102
LP(2)O25	1.86423	σ*(C27-N29)	0.06965	25.29	0.69	0.120
LP(1)N29	1.76918	π*(O25=C27)	0.25959	39.96	0.34	0.104

Table S6 Reactivity descriptors as Fukui functions (f_k^+, f_k^-), local softness (s_k^+, s_k^-), local electrophilicity indices (ω_k^+, ω_k^-) for NIC-OXA salt using Hirshfeld atomic charges.

**Atom no.**	f_k^+	s_k^+	ω_k^+	**Atom no.**	f_k^-	s_k^-	ω_k^-
O9	0.1067	0.0200	0.2174	C18	0.0581	0.0109	0.1184
O25	0.1067	0.0200	0.2174	C34	0.0581	0.0109	0.1184
O1	0.0738	0.0138	0.1505	C19	0.0577	0.0108	0.1177
O4	0.0738	0.0138	0.1505	C35	0.0577	0.0108	0.1177
N13	0.0341	0.0064	0.0695	O9	0.0461	0.0086	0.0939
N29	0.0341	0.0064	0.0695	O25	0.0461	0.0086	0.0939
O2	0.0318	0.0060	0.0648	C12	0.0386	0.0072	0.0786
O5	0.0318	0.0060	0.0648	C28	0.0386	0.0072	0.0786
C3	0.0299	0.0056	0.0609	N7	0.0340	0.0064	0.0694
C6	0.0299	0.0056	0.0609	N23	0.0340	0.0064	0.0694
